# Targeted Resequencing and Analysis of the Diamond-Blackfan Anemia Disease Locus *RPS19*

**DOI:** 10.1371/journal.pone.0006172

**Published:** 2009-07-09

**Authors:** Alvaro Martinez Barrio, Oskar Eriksson, Jitendra Badhai, Anne-Sophie Fröjmark, Erik Bongcam-Rudloff, Niklas Dahl, Jens Schuster

**Affiliations:** 1 The Linnaeus Centre for Bioinformatics Uppsala University/Swedish University of Agricultural Sciences, Uppsala University, Uppsala, Sweden; 2 Department of Genetics and Pathology, The Rudbeck Laboratory, Uppsala University, Uppsala, Sweden; 3 Department of Animal Breeding and Genetics, Uppsala University, Uppsala, Sweden; Aarhus University, Denmark

## Abstract

**Background:**

The Ribosomal protein S19 gene locus (*RPS19*) has been linked to two kinds of red cell aplasia, Diamond-Blackfan Anemia (DBA) and Transient Erythroblastopenia in Childhood (TEC). Mutations in *RPS19* coding sequences have been found in 25% of DBA patients, but not in TEC patients. It has been suggested that non-coding *RPS19* sequence variants contribute to the considerable clinical variability in red cell aplasia. We therefore aimed at identifying non-coding variations associated with DBA or TEC phenotypes.

**Methodology/Principal Findings:**

We targeted a region of 19'980 bp encompassing the *RPS19* gene in a cohort of 89 DBA and TEC patients for resequencing. We provide here a catalog of the considerable, previously unrecognized degree of variation in this region. We identified 73 variations (65 SNPs, 8 indels) that all are located outside of the *RPS19* open reading frame, and of which 67.1% are classified as novel. We hypothesize that specific alleles in non-coding regions of *RPS19* could alter the binding of regulatory proteins or transcription factors. Therefore, we carried out an extensive analysis to identify transcription factor binding sites (TFBS). A series of putative interaction sites coincide with detected variants. Sixteen of the corresponding transcription factors are of particular interest, as they are housekeeping genes or show a direct link to hematopoiesis, tumorigenesis or leukemia (e.g. GATA-1/2, PU.1, MZF-1).

**Conclusions:**

Specific alleles at predicted TFBSs may alter the expression of *RPS19*, modify an important interaction between transcription factors with overlapping TFBS or remove an important stimulus for hematopoiesis. We suggest that the detected interactions are of importance for hematopoiesis and could provide new insights into individual response to treatment.

## Introduction

Diamond Blackfan Anemia (DBA) is a congenital pure red cell aplasia (OMIM 205900) typically presenting within the first year of life [1], [2]. The gene encoding ribosomal protein S19 (*RPS19*) [3], [4] has been shown to be mutated in 25% of DBA patients [5]. Recently, mutations in several other ribosomal protein genes have been identified in approximately 10% of DBA patients [6]–[9].

Transient erythroblastopenia of childhood (TEC; OMIM 227050) is a transient red cell aplasia with clinical similarities to DBA [10], [11]. Linkage analysis has indicated an association between TEC and the region encompassing *RPS19* but no structural mutations have been identified so far [12].

Until now, more than 70 *RPS19* mutations have been reported in DBA patients [5]. Mutations are spread out over the entire gene, including non-sense and mis-sense mutations as well as deletions and insertions. DBA is characterized by a marked clinical heterogeneity without correlations to any specific mutation [2], [13]. At least 30% of DBA patients respond to steroid treatment and patients carrying *RPS19* mutations display a poorer response [5]. The marked clinical heterogeneity strongly implies the involvement of genetic and/or environmental tissue specific modulators [2]. It has been suggested that the red cell aplasia is caused by ribosomal protein haploinsufficiency. Consequently, the expression level of a specific ribosomal protein becomes critical for the disease. The expression may be influenced by non-coding variations resulting in a decrease in the amount of protein available below a critical threshold [14]. Moreover, the clinical variability associated with a mutation in a specific structural ribosomal protein gene may be related to non-coding variants on the non-mutant allele. Studies so far have focused on protein coding parts and no detailed catalog of non-coding genetic variation is available. The identification of putative regulatory sequence elements in non-coding regions (such as transcription factor binding sites) is therefore of importance for future research. Many transcription factors (TFs) have been implicated in human disorders, for example HNF4alpha in diabetes [15], [16], USF1 in familial combined hyperlipidemia [17] and AP2alpha in cleft palate [18].

We therefore aimed at identifying non-coding variations that are associated with the DBA or TEC phenotypes. Here, we report on the targeted resequencing of the entire *RPS19* locus in 77 DBA and 12 TEC patients not carrying a mutation in exons of the *RPS19* gene and we provide a catalog of the genetic variations identified. Furthermore, we searched the entire region for putative transcription factor binding sites (TFBS) some of which are presumably altered by the variations identified. We suggest that gene variants at TFBSs influence the expression of *RPS19* with a resulting effect on disease pattern and response to treatment. These findings are important to clarify the regulation of *RPS19* gene expression and for our understanding of the pathobiology behind DBA.

## Results

### Genetic catalog of the *RPS19* locus

**Figure 1:**
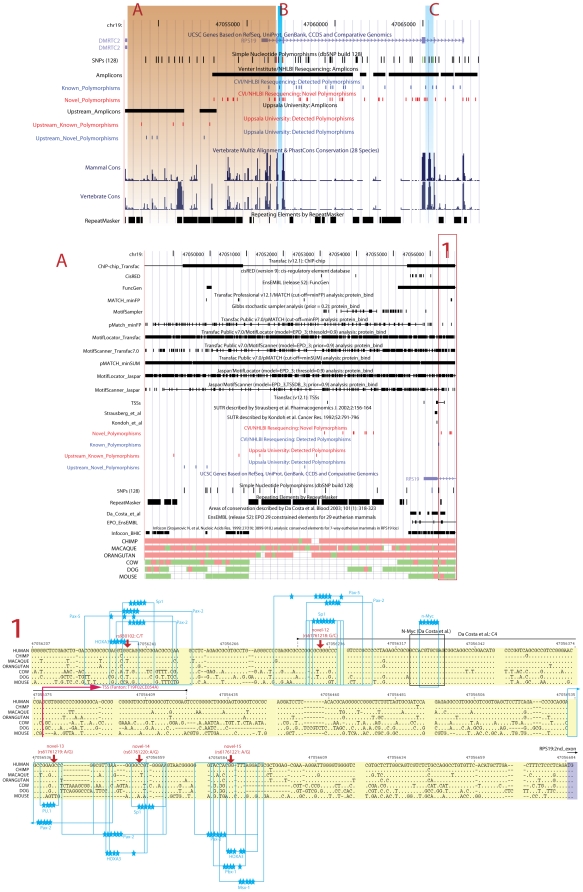
Schematic view of the *RPS19* locus on chromosome 19. The genomic region targeted by the resequencing analysis (chr19:47'048'100–47'068'200) is shown as a snap shot using the UCSC Genome Browser (http://www.genome.ucsc.edu/). Amplicons and mammalian conservation are indicated, as well as detected variations (novel and known Polymorphisms, respectively). SNPs contained in dbSNP (version 128) are shown next to our reported variants. The six exons of the *RPS19* gene and 3′-end of the DMRTC2 gene located upstream are shown in grey. A more detailed view presenting all available information compiled on the targeted region is shown in supplementary figure S2. The whole analyzed region encompasses 19'980 kbp. (A) Detailed picture of the overlapping TFBSs and functional data extracted from EnsEMBL and Transfac (supplementary text S2) next to discovered variation in the upstream area towards the DMRCT2 gene. A more detailed section (1) describes the multiple alignment of a region comprising 476 bp upstream of the RPS19 Start codon (ATG) located in the second exon. For the 7 species selected, five different SNPs (in red with red arrows pointing the SNP position in the sequence), a transcription start site (TSS) from the Fantom database (presented as an arrow indicating transcriptional direction), several interesting TFBSs overlapping highly conserved SNPs (in blue with blue stars indicating important positions; table 2), and the four highly conserved regions reported by DaCosta *et al*. (containing the detected n-Myc motif) are highlighted.

We initially targeted a region of 14.7 kbp on human chromosome 19 (chr19:47,053,043–47,068,080), encompassing *RPS19* and 2 kbp of flanking region (Accession numbers: RSG_JCVI|RPS19-004110_004111-C_G, RSG_JCVI|RPS19-005767_005771-D_CTAA). Variations were assigned in all individuals to provide a genetic map of the *RPS19* locus. In addition, we analyzed a region upstream of the initial sequencing effort in a subset of patients (chr19:47,048,100–47,053,043); both analyzed regions together comprised of 19'980 bp (figure 1). We detected a total of 73 variations of which 65 were single nucleotide polymorphisms (SNPs; 89.1%) and 8 were insertion/deletion variants (indels; 10.9%). Forty-three SNPs (66.2%) and 6 indels (75.0%) were not previously described and could be classified as novel (figure 1 and table 1; SNPs identified in this study are referred to as “novel” throughout this report - their subsequently assigned database identifiers are listed in table 1). Altogether, 49 of the variations identified (67.1%) were novel. One of the novel indels overlaps with a known SNP (rs725332; table 1). All variants are located outside of the protein coding sequence of *RPS19*. Interestingly, the density of variations (SNP or indel) is one per 273.7 bp (3.6 variations kbp^−1^), including one SNP per 293.8 bp (3.4 SNPs kbp^−1^). This is in contrast to the expected density of one SNP per 1.9 to 2.18 kbp^−1^ that has been estimated for human chromosome 19 in previous studies [19], [20]. Several of the detected SNPs show a high frequency within the patient material (table 1). However, a considerable number of variations (30 out of 73) show frequencies of less than 1% and the prevalence of these “private” or rare variations is high, compared to previous estimates of 7% [21].

**Table 1:** Variation detected in the DBA/TEC patient cohort within the resequenced region on chromosome 19.

SNP ID^a^	Alleles^b^	Position on Chr19^c^		#Homozygous^d^	#Heterozygous^d^	Frequency within cohort^e^	#Chromosomes analyzed^e^	Reference Allele^c^	dbSNP ID^f^
		START	END						
1	A/g	47049015	47049015	0	1	0.026	38	A	**novel-1**
2	A/c	47049295	47049295	4	7	0.39	38	A	rs4803512
3	T/c	47049909	47049909	4	8	0.42	38	T	rs6509002
4	CT/-	47049621	47049622	1	0	0.09	22	CT	rs3214574
5	CTTCTT/-	47051353	47051358	30	35	0.62	158	CTTCTT	**novel-2** *(overlapping rs7253322)*
6	T/c	47050793	47050793	1	0	0.09	22	T	**novel-3**
7	G/c	47052575	47052575	3	8	0.368	38	G	rs7258162
8	G/t	47052894	47052894	4	9	0.447	38	T	**novel-4**
9	A/g	47053121	47053121	19	22	0.5660	106	G	**novel-5 (rs58857981)**
10	G/t	47053622	47053622	0	2	0.0185	108	G	**novel-6 (rs61761212)**
11	A/g	47053901	47053901	0	1	0.0075	134	A	**novel-7 (rs61761213)**
12	A/g	47053920	47053920	0	1	0.0077	130	A	**novel-8 (rs61761214)**
13	T/c	47054215	47054215	0	2	0.0122	164	T	**novel-9 (rs61761215)**
14	C/t	47054248	47054248	0	2	0.0061	164	C	**novel-10 (rs61761216)**
15	C/t	47054449	47054449	0	2	0.0159	126	C	**novel-11 (rs61761217)**
16	C/t	47056235	47056235	25	47	0.5640	172	T	rs930102:C
17	C/g	47056293	47056293	0	1	0.0057	176	C	**novel-12 (rs61761218)**
18	G/a	47056542	47056542	1	0	0.0110	182	G	**novel-13 (rs61761219)**
19	G/a	47056557	47056557	1	0	0.0110	182	G	**novel-14 (rs61761220)**
20	G/a	47056585	47056585	1	0	0.0110	182	G	**novel-15 (rs61761221)**
21	CC/c	47056836	47056836	29	44	0.5667	180	C	**novel-16 (rs56182210)**
22	C/g	47056844	47056844	30	0	0.6522	92	G	rs2075749:C
23	T/-	47057012	47057012	0	1	0.0054	184	T	**novel-17 (rs61761223)**
24	C/g	47057167	47057167	0	1	0.0056	178	C	**novel-18 (rs61761226)**
25	A/g	47057784	47057784	27	36	0.5844	154	G	rs12461131:A
26	T/c	47057804	47057804	27	37	0.5833	156	C	rs12461099:T
27	-/ctaa	47057950	47057953	23	37	0.5608	148	CTAA	rs34598858:C/-
28	G/a	47057955	47057955	0	1	0.0135	74	G	**novel-19 (rs61761228)**
29	A/g	47058032	47058032	0	1	0.0135	74	A	**novel-20 (rs61761229)**
30	A/g	47058072	47058072	27	4	0.8056	72	G	rs3786539:A
31	G/t	47058087	47058087	23	0	0.6216	74	T	rs3786538:G
32	T/a	47058376	47058376	0	1	0.0217	46	T	**novel-21 (rs61761230)**
33	G/a	47058432	47058432	0	3	0.0375	80	G	**novel-22 (rs61761231)**
34	A/t	47059461	47059461	23	30	0.6032	126	T	rs7250787:A
35	C/t	47059746	47059746	30	48	0.5870	184	T	rs873282:C
36	G/a	47059747	47059747	0	1	0.0054	184	G	**novel-23 (rs61761232)**
37	G/a	47059817	47059817	0	1	0.0054	184	G	**novel-24 (rs61761233)**
38	A/g	47059856	47059856	23	49	0.5163	184	G	rs12972552:A
39	T/g	47059922	47059922	0	1	0.0054	184	T	**novel-25 (rs61761234)**
40	G/t	47060250	47060250	0	1	0.0056	178	G	**novel-26 (rs61761235)**
41	C/cccacc	47060402	47060402	0	3	0.0163	184	C	**novel-27 (rs61761236)**
42	T/c	47060414	47060414	0	1	0.0056	178	T	**novel-28 (rs61761237)**
43	A/g	47060469	47060469	47	34	0.7273	176	G	rs12974044:A
44	G/a	47060578	47060578	29	48	0.5824	182	A	rs7254214:G
45	C/t	47061583	47061583	29	48	0.5761	184	T	rs7259596:C
46	G/c	47061654	47061654	0	1	0.0054	184	G	**novel-29 (rs61761238)**
47	G/gg	47062448	47062448	0	1	0.0054	184	G	**novel-30 (rs61761239)**
48	C/t	47062504	47062504	0	1	0.0055	182	C	**novel-31 (rs61761240)**
49	G/a	47062690	47062690	0	1	0.0054	184	G	**novel-32 (rs61761241)**
50	G/c	47062807	47062807	0	1	0.0054	184	G	rs11879132:A
51	A/g	47064006	47064006	0	1	0.0076	132	A	**novel-33 (rs61761242)**
52	A/c	47064297	47064297	22	29	0.5703	128	C	rs3786536:A
53	G/t	47064380	47064380	0	1	0.0055	182	G	**novel-34 (rs61761243)**
54	C/g	47064567	47064567	0	2	0.0345	58	C	**novel-35 (rs61761244)**
55	T/a	47064585	47064585	0	2	0.0114	176	T	**novel-36 (rs61762288)**
56	C/a	47064639	47064639	0	1	0.0057	176	C	**novel-37 (rs61762289)**
57	C/t	47064647	47064647	0	1	0.0057	176	C	**novel-38 (rs61762290)**
58	C/t	47064688	47064688	0	1	0.0057	176	C	**novel-39 (rs61762291)**
59	C/a	47064772	47064772	0	1	0.0057	176	C	**novel-40 (rs61762292)**
60	A/g	47065138	47065138	30	45	0.5769	182	G	rs1366610:A
61	G/c	47065142	47065142	0	1	0.0054	184	G	**novel-41 (rs61762294)**
62	G/t	47065190	47065190	1	0	0.0109	184	G	**novel-42 (rs61762295)**
63	T/c	47065519	47065519	29	45	0.5920	174	C	rs2075750:T
64	G/a	47065675	47065675	0	1	0.0056	180	G	**novel-43 (rs61762296)**
65	T/c	47065733	47065733	48	36	0.7333	180	C	rs2075751:T
66	G/c	47066171	47066171	0	2	0.0109	184	G	**novel-44 (rs61762297)**
67	T/c	47066237	47066237	0	1	0.0054	184	T	**novel-45 (rs61762298)**
68	G/t	47066676	47066676	30	47	0.5879	182	T	rs2075752:G
69	T/c	47067084	47067084	31	3	0.6915	94	C	rs2075754:T
70	C/-	47067232	47067232	0	2	0.0109	184	C	**novel-46 (rs61762299)**
71	C/a	47067432	47067432	0	1	0.0054	184	C	**novel-47 (rs61762300)**
72	G/a	47067568	47067568	0	1	0.0055	182	G	**novel-48 (rs61762301)**
73	G/a	47067955	47067955	0	1	0.0055	182	G	**novel-49 (rs61762302)**

a variant identifier.

b Most frequent (Major) and alternative (minor) allele within our patient cohort.

c as compared to the human reference genome (hg18, build 36.1).

d number of heterozygous and homozygous cases, respectively, within our patient cohort.

e allele frequency and sample size (number of analyzed chromosomes), excluding patient samples with undetermined genotype (NN).

f database identifier (dbSNP128) for previously described SNP (http://www.ncbi.nlm.nih.gov/SNP/) or number of novel SNP with subsequently assigned database identifier (dbSNP129) in brackets, respectively.

### Comparative genomic sequence analysis of the *RPS19* locus

Human *RPS19* has homologs in eukaryotes and archaebacteria but no eubacterial counterparts [22]. RPS19 is a component of the 40S subunit of the ribosome, which is important for regulation of translation of mRNAs into polypeptides [23]. From all the mammalian sequences available, we selected those assembled into chromosomes with high coverage and lack of gaps in the targeted genomic region, assuring gene synteny and that the original structure of the human *RPS19* gene is conserved (i.e. number and order of exons). We obtained and aligned syntenic genomic regions of 200 kb around the orthologous *RPS19* gene from 6 species (mouse, dog, cow, orangutan, macaque and chimpanzee; supplementary figure S1 and supplementary text S1). Infocon [24] identified a total of 161 blocks of high information content (BHICs) with highly conserved multi-species alignments within the 200 kb region. They averaged 20 bp in size and their distribution in protein coding, non-coding and untranslated regions is shown in supplementary figure S2. A high information content block is a cluster of conservation between species where the alignment contains information for every species represented. Because this alignment is so highly conserved at almost every position, the consensus sequence for each BHIC defaults to the reference genome used in our alignment. 12 SNPs were contained in BHICs, 10 of them are detailed in supplementary table S1. If we consider the conservation of the polymorphic nucleotide, 7 of them are totally conserved across species (novel-12, novel-14, novel-15, novel-17, novel-18, rs2075749, rs2075750), 3 present a miss-match in one of the species (novel-13, mouse; novel-40, dog; rs1366610, mouse) and another 3 present two miss-matches (novel-3, cow-dog; novel-42, cow-mouse; rs930102, human-cow). With this analysis, we discovered that in many cases the human variation is found across species. Additionally, we downloaded the 29-way eutherian mammals Enredo-Pecan-Ortheus (EPO) alignment track containing ultra-conserved elements from the EnsEMBL database and compared this to our alignment results (supplementary figure S1). From nine conserved elements defined as EPO, five were entirely contained in our 7-way species alignment. Surprisingly, two were not contained at all. None of the novel SNPs was contained in a defined EPO region.

All information obtained in our report and from external resources was converted into *.gff files in an effort towards improving the annotation of the *RPS19* locus (*.gff files can be imported to the UCSC genome browser for visualization and are compiled in supplementary text S2; see also figure S2).

### Identified putative transcription factor binding sites that superimpose with novel SNPs

We used the resulting multi-species alignment to analyze whether any of the detected variation marks out any sequence element important for regulation by modulators or regulating factors. We searched selected genomic regions for putative transcription factor binding sites (TFBS) focusing on regions with a high degree of conservation (figure 1). Our aim was to identify whether any of the detected SNPs coincide with predicted TFBS. A number of detected variations fall within putative TFBS (supplementary table S1). Additionally, to further narrow down the number of identified TFBS, we asked whether the identified TFBS are likely to be functionally relevant for adaptations in expression of RPS19 protein in the context of DBA/TEC. We searched the literature for association of the corresponding transcription factors to general transcription, tumorigenesis and hematopoiesis. Sixteen different transcription factors (GATA-1 and -2, CDC5, Ebox, HOXA3, MSX-1, MZF1, PAX-2, -5 and -6, PBX-1, PPARalpha, PPARgamma, PU.1, SP1, YY1) are of particular interest with possible link to the DBA and TEC phenotypes (e.g. important for hematopoiesis, implicated in cancer development, strong general transcription factor). The putative TFBS coincide with 23 of the detected variants (table 2). The corresponding transcription factors bind to 15 possible sites upstream of the *RPS19* coding region (TFBS encompassing 19 SNPs), at one position within the second intron (one detected SNP) and to three sites located in intron 4 (three SNPs). In some cases, the TFBS was independently identified by two different tools/databases (e.g. PBX-1 binding site in upstream region; table 2).

**Table 2:** Putative TFBSs overlaying detected SNPs#.

Region^a^	SNP^b^	Ref SNP^c^	TFBS name^d^	source^e^	Motif^f^	Ref allele score^g^	Non-ref allele score^h^	Tool^i^	MCS^j^
					Position (chr:start-end:strand)				
**upstream**	**novel-1**	[G/A]	YY1 (Yin and Yang 1)	Transfac:v7.0:M00059	taatCCCAGc[G/a]ctttgg	-	0.996/0.976	pMATCH (minSUM)	(N|N)
					19:47049005-47049021:+1				
			Pax-2 (Paired box 2)	Transfac:v7.0:M00098	aatcCCAGC[g/a]ctttgggag	0.992/0.943	-		(N|N)
					19:47049006-47049024:+1				
			Pax-2	Transfac:v7.0:M00098	atccCAGC[G/A]ctttgggagg	0.918/0.940	0.910/0.936		(N|N)
					19:47049007-47049025:+1				
			HOXA3 (Homeobox cluster protein)	Transfac:v7.0:M00395	AGC[G/A]Ctttg	1.000/0.963	-		(N|N)
					19:47049012-47049020:+1				
			HOXA3	Transfac:v7.0:M00395	C[G/A]CTTtggg	0.940/0.941	0.956/0.953		(N|N)
					19:47049014-47049022:+1				
**upstream**	**rs4803512**	[A/C]	Pax-2	Transfac:v7.0:M00098	tgaaCCATTc[a/c]gtacatag	0.982/0.922	0.982/0.919	pMATCH (minSUM)	(N|N)
					19:47049285-47049303:+1				
			HOXA3	Transfac:v7.0:M00395	ACCATtc[a/c]g	0.954/0.948	0.954/0.948		(N|N)
					19:47049288-47049296:+1				
			Pax-2	Transfac:v7.0:M00098	accaTTC[A/C]Gtacataggaa	0.947/0.891	-		(N|N)
					19:47049288-47049306:+1				
			Pax-2	Transfac:v7.0:M00098	ccatTC[A/C]Gtacataggaaa	0.975/0.903	-		(N|N)
					19:47049289-47049307:+1				
			HOXA3	Transfac:v7.0:M00395	CATTC[a/c]gta	0.970/0.946	-		(N|N)
					19:47049290-47049298:+1				
			HOXA3	Transfac:v7.0:M00395	TTC[A/C]Gtaca	1.000/0.975	-		(N|N)
					19:47049292-47049300:+1				
			Pax-2	Transfac:v7.0:M00098	ttc[a/c]GTACAtaggaaaacc	0.981/0.946	0.981/0.945		(N|N)
					19:47049292-47049310:+1				
			HOXA3	Transfac:v7.0:M00395	[A/C]GTACatag	-	0.957/0.961		(N|N)
					19:47049295-47049303:+1				
			Msx-1 (Msh-like homeobox protein 1)	Transfac:v7.0:M00394	c[a/c]gTACATa	1.000/1.000	1.000/1.000		(N|N)
					19:47049294-47049302:+1				
**upstream**	**rs3214574**	[-/CT]	Pax-2	Transfac:v7.0:M00098	cttcCCTCCccttag[-/ct]at	0.924/0.893	0.924/0.894	pMATCH (minSUM)	(N|N)
					19:47049605-47049623:+1		(cttcCCTCCccttagataa)		
			HOXA3	Transfac:v7.0:M00395	CCCCTtag[-/ct]	0.926/0.942	-		(N|N)
					19:47049612-47049620:+1				
			HOXA3	Transfac:v7.0:M00395	CCTTAg[-/ct]a	0.987/0.942	0.987/0.959		(N|N)
					19:47049614-47049622:+1		(CCTTAgata)		
			Msx-1	Transfac:v7.0:M00394	cctTAG[-/CT]a	0.955/0.933	0.962/0.938		(N|N)
					19:47049614-47049622:+1		(cctTAGATa)		
			HOXA3	Transfac:v7.0:M00395	CTTAG[-/ct]at	0.957/0.943	-		(N|N)
					19:47049615-47049623:+1				
			Pax-2	Transfac:v7.0:M00098	ag[-/ct]ATAACttagatatat	0.972/0.897	0.972/0.896		(N|N)
					19:47049618-47049636:+1		(ttagATAACttagatatat,		
							19:47049616-47049634:+1)		
			Pax-6	Transfac:v7.0:M00097	cctccCCTTAgataacttaga	-	0.987/0.976	pMATCH	(N|N)
					19:47049609-47049629:+1			(minSUM, minFP)	
			HOXA3	Transfac:v7.0:M00395	[-/ct]ATAACttag	1.000/0.995	1.000/0.995		(N|N)
					19:47049622-47049630:+1				
			GATA-1 (GATA-binding factor 1)	Transfac:v7.0:M00346	ttaGATAAct	-/0.9	1629.46/0.9	MotifScanner	(N|N)
					19:47049616-47049625:+1				
			GATA-2 (GATA-binding factor 2)	Transfac:v7.0:M00082	ttaGATAAct	-/0.9	3124.5/0.9		(N|N)
					19:47049616-47049625:+1				
			Cdc5 (Cdc5 cell division control protein 5)	Transfac:v7.0:M00478	G[-/ct]aTAacttag	3343.59/0.9	-/0.9		(N|N)
					19:47049619-47049630:+1				
**upstream**	**rs6509002**	[C/T]	Pax-2	Transfac:v7.0:M00098	aggaGTAGActagagg[c/t]ca	0.915/0.916	0.915/0.916	pMATCH	(y+| N)
					19:47049893-47049911:+1			(minSUM)	
			Pax-2	Transfac:v7.0:M00098	agtaGACTAgagg[c/t]cacct	0.840/0.906	0.840/0.906		(y|N)
					19:47049896-47049914:+1				
			Pax-2	Transfac:v7.0:M00098	agagG[c/T]CACctctccttca	0.992/0.914	-		(y−|N)
					19:47049904-47049922:+1				
			PPARalpha (PPAR:RXR heterodimers)	Transfac:v12.1:M00242	aAggAgTAGactAgAGG[c/T]CA	0.784/0.784	-	MATCH	(y+| N)
					19:47049892-47049911:+1			(minFP)	
			PPARgamma	Transfac:v7.0:M00528	gagtaGactAgaGg[c/T]ca	3331.06/0.9	-/0.9	MotifScanner	(y|N)
					19:47049895-47049911:+1				
**upstream**	**novel-3**	[C/T]	Pax-2	Transfac:v7.0:M00098	cagaGTTCCcta[c/t]gttccc	-	0.963/0.919	pMATCH	(Y|N)
					19:47050781-47050799:+1			(minSUM)	
			Pax-2	Transfac:v7.0:M00098	agagTTCCCta[c/t]gttccca	0.982/0.911	0.982/0.899		(Y|N)
					19:47050782-47050800:+1				
			Msx-1	Transfac:v7.0:M00394	cccTA[C/T]GTt	0.955/0.940	0.992/0.965		(Y|N)
					19:47050788-47050796:+1				
			HOXA3	Transfac:v7.0:M00395	CCTA[C/T]gttc	0.987/0.943	1.000/0.945		(Y|N)
					19:47050789-47050797:+1				
			Pax-2	Transfac:v7.0:M00098	cta[c/t]GTTCCcaaggggcca	0.963/0.928	0.963/0.928		(y+|N)
					19:47050790-47050808:+1				
			PPARgamma2 (peroxisome proliferator-	Transfac:v12.1:M00515	ctcTcaGgCAgaGTtcCCtA[C/t]gT	0.604/0.675	0.604/0.682	MATCH	(y+|N)
			activated receptor gamma)		19:47050773-47050795:+1			(minFP)	
**upstream**	**rs7258162**	[C/G]	Pax-2	Transfac:v7.0:M00098	gcgcCCACCacct[c/g]cccca	0.992/0.947	0.992/0.921	pMATCH	(N|N)
					19:47052562-47052580:+1			(minSUM)	
			Msx-1	Transfac:v7.0:M00394	cacCACCT[c/g]	0.906/0.936	-		(N|N)
					19:47052567-47052575:+1				
			HOXA3	Transfac:v7.0:M00395	ACCACct[c/g]c	1.000/0.941	-		(N|N)
					19:47052568-47052576:+1				
			Pax-2	Transfac:v7.0:M00098	t[c/g]ccCCAGCtaatgttttg	0.992/0.930	0.992/0.929		(N|N)
					19:47052574-47052592:+1				
			Ebox (E-box)	Transfac:v12.1:M01034	cCACCT[c/G]cCc	0.998/0.996	-	MATCH	(N|N)
					19:47052569-47052578:+1			(minFP)	
**upstream**	**novel-4**	[G/T]	Pax-2	Transfac:v7.0:M00098	tatcCCACTgttt[G/t]taggt	-	1.000/0.900	pMATCH	(N|N)
					19:47052881-47052899:+1			(minSUM)	
			Msx-1	Transfac:v7.0:M00394	cacTGTTT[G/t]	-	0.951/0.959		(N|N)
					19:47052886-47052894:+1				
			Pax-2	Transfac:v7.0:M00098	cactGTTT[G/T]taggtactac	0.971/0.951	0.974/0.952		(N|N)
					19:47052886-47052904:+1				
			Pax-2	Transfac:v7.0:M00098	[g/t]tagGTACTacagcctcaa	0.903/0.899	0.903/0.898		(N|N)
					19:47052894-47052912:+1				
**upstream**	**novel-5**	[A/G]	Pax-2	Transfac:v7.0:M00098	gtatGATTCcaactat[a/g]tg	0.992/0.935	0.992/0.923	pMATCH	(N|N)
					19:47053105-47053123:+1			(minSUM)	
			Msx-1	Transfac:v7.0:M00394	aacTAT[A/G]Tg	0.909/0.931	0.917/0.936		(N|N)
					19:47053115-47053123:+1				
			Pbx-1	Transfac:v7.0:M00096	actAT[A/G]TGa	0.964/0.944	1.000/0.971		(N|N)
					19:47053116-47053124:+1				
			Pax-2	Transfac:v7.0:M00098	tat[a/g]TGACAttctggaaaa	0.929/0.902	0.929/0.903		(N|N)
					19:47053118-47053136:+1				
			Pax-2	Transfac:v7.0:M00098	at[a/g]tGACATtctggaaaaa	0.992/0.899	0.992/0.912		(N|N)
					19:47053119-47053137:+1				
			HOXA3	Transfac:v7.0:M00395	[A/g]TGACattc	-	0.967/0.962		(N|N)
					19:47053121-47053129:+1				
**upstream**	**novel-6**	[T/G]	Pax-2	Transfac:v7.0:M00098	agtaCCA[T/G]Ctactcgacag	0.992/0.909	0.974/0.899	pMATCH	(N|N)
					19:47053615-47053633:+1			(minSUM)	
			HOXA3	Transfac:v7.0:M00395	[t/G]CTACtcga	0.987/0.964	-		(N|N)
					19:47053622-47053630:+1				
			Pax-2	Transfac:v7.0:M00098	[t/g]actcGACAGgctgaggtag	-	0.972/0.983	pMATCH	(N|N)
					19:47053623-47053641:+1			(minFP)	
**upstream**	**novel-7**	[G/A]	HOXA3	Transfac:v7.0:M00395	AAA[g/A]Ctaac	0.984/0.975	-	pMATCH	(N|N)
					19:47053898-47053906:+1			(minSUM)	
			Pax-2	Transfac:v7.0:M00098	a[g/a]ctAACATgtacaaaaat	0.899/0.922	0.899/0.923		(N|N)
					19:47053900-47053918:+1				
			Msx-1	Transfac:v7.0:M00394	a[g/a]cTAACAt	0.949/0.940	0.949/0.940		(N|N)
					19:47053900-47053908:+1				
			HOXA3	Transfac:v7.0:M00395	[g/A]CTAAcatg	0.951/0.944	-		(N|N)
					19:47053901-47053909:+1				
			Msx-1	Transfac:v7.0:M00394	[g/a]ctAACATg	0.962/0.967	0.962/0.957		(N|N)
					19:47053901-47053909:+1				
			HOXA3	Transfac:v7.0:M00395	[G/a]CTAAcatg	-	1.000/0.982		(N|N)
					19:47053901-47053909:+1				
**upstream**	**novel-8**	[G/A]	Pax-2	Transfac:v7.0:M00098	acatGTACAaaaatt[g/a]aca	0.915/0.904	0.915/0.907	pMATCH	(N|N)
					19:47053905-47053923:+1			(minSUM)	
			HOXA3	Transfac:v7.0:M00395	[G/A]ACAAAaatt	0.973/0.974	0.973/0.974		(N|N)
					19:47053911-47053919:+1				
			Msx-1	Transfac:v7.0:M00394	caaAAATT[g/a]	-	0.989/0.978		(N|N)
					19:47053912-47053920:+1				
			Pbx-1a (homeo domain factor Pbx-1)	Transfac:v7.0:M00096	aaaAATT[g/A]a	0.983/0.955	-		(N|N)
					19:47053913-47053921:+1				
			HOXA3	Transfac:v7.0:M00395	AAAATt[g/a]ac	0.970/0.964	0.970/0.964		(N|N)
					19:47053914-47053922:+1				
			Msx-1	Transfac:v7.0:M00394	aaaTT[g/A]ACa	0.913/0.931	-		(N|N)
					19:47053915-47053923:+1				
			Msx-1	Transfac:v7.0:M00394	aatT[g/A]ACAc	0.949/0.948			(N|N)
					19:47053916-47053924:+1				
			Pax-2	Transfac:v7.0:M00098	aatt[G/a]ACACcagggacaca	-	1.000/0.920		(N|N)
					19:47053916-47053934:+1				
**upstream**	**novel-9**	[C/T]	Msx-1	Transfac:v7.0:M00394	tgcCTTA[c/T]g	0.917/0.943	-	pMATCH	(N|N)
					19:47054208-47054216:+1			(minSUM)	
			HOXA3	Transfac:v7.0:M00395	CCTTA[c/t]gtc	-	0.987/0.953		(N|N)
					19:47054210-47054218:+1				
			Msx-1	Transfac:v7.0:M00394	cctTA[C/T]GTc	0.955/0.933	0.992/0.958		(N|N)
					19:47054210-47054218:+1				
			HOXA3	Transfac:v7.0:M00395	CTTA[C/T]gtcc	0.970/0.959	0.957/0.948		(N|N)
					19:47054211-47054219:+1				
			Msx-1	Transfac:v7.0:M00394	a[c/t]gTCCATc	0.902/0.933	0.902/0.933		(N|N)
					19:47054214-47054222:+1				
			Pbx-1b (homeo domain factor Pbx-1)	Transfac:v7.0:M00124	ta[c/t]gTCcATCAAaac	2253.81/0.9	10900.5/0.9	MotifScanner	(N|N)
					19:47054213-47054227:+1				
			Pbx1	Jaspar:v3.0:MA0070	a[c/t]gTCcATCAaa	2378.14/0.9	8330.43/0.9		(N|N)
					19:47054214-47054225:+1				
**upstream**	**novel-10**	[T/C]	Pax-6	Transfac:v7.0:M00097	ctctgCTTTAtataaatttta[t/c]	0.948/0.968	0.948/0.968	pMATCH	(N|N)
					19:47054227-47054247:+1			(minSUM)	
			Pax-2	Transfac:v7.0:M00098	ataaATTTTa[t/c]ctaagctt	0.982/0.932	0.982/0.941		(N|N)
					19:47054238-47054256:+1				
			Pax-2	Transfac:v7.0:M00098	ttta[T/C]CTAAgcttcactct	0.924/0.907	0.949/0.922		(N|N)
					19:47054244-47054262:+1				
			Msx-1	Transfac:v7.0:M00394	a[t/c]cTAAGCt	0.989/0.968	0.989/0.967		(N|N)
					19:47054247-47054255:+1				
			HOXA3	Transfac:v7.0:M00395	[T/C]CTAAgctt	1.000/0.955	0.951/0.946		(N|N)
					19:47054248-47054256:+1				
**upstream**	**novel-11**	[T/C]	HOXA3	Transfac:v7.0:M00395	CCTGTaatc[t/c]	0.970/0.958	0.970/0.958	pMATCH	(N|N)
					19:47054440-47054448:+1			(minSUM)	
			Pax-2	Transfac:v7.0:M00098	gtaaTC[T/c]CAgcattttggg	-	0.989/0.931		(N|N)
					19:47054443-47054461:+1				
			Pax-2	Transfac:v7.0:M00098	aatc[T/C]CAGCattttgggag	0.992/0.952	0.966/0.937		(N|N)
					19:47054445-47054463:+1				
			Pax-2	Transfac:v7.0:M00098	atc[t/c]CAGCAttttgggagg	0.918/0.927	0.918/0.927		(N|N)
					19:47054446-47054464:+1				
**upstream**	**rs930102**	[C/T]	Pax-2	Transfac:v7.0:M00098	cgagG[c/T]GGCagggccggac	0.914/0.926	-	pMATCH	(Y|N)
					19:47056230-47056248:+1			(minSUM)	
			HOXA3	Transfac:v7.0:M00395	CGAGG[c/t]ggc	0.973/0.979	-		(Y|N)
					19:47056230-47056238:+1				
			Pax-2	Transfac:v7.0:M00098	agg[c/t]GGCAGggccggacgc	0.887/0.901	0.887/0.902		(Y|N)
					19:47056232-47056250:+1				
			Sp1 (stimulating protein 1)	Transfac:v7.0:M00008	g[c/t]GGCAGggc	1.000/0.976	1.000/0.976		(Y|N)
					19:47056234-47056243:+1				
			Pax-5 (B-cell-specific activating protein)	Transfac:v7.0:M00143	accgggcgCgagG[c/t]gGcagggccggacg	2609.34/0.9	1057.56/0.9	MotifScanner	(y+|N)
					19:47056222-47056249:+1				
**upstream**	**novel-12**	[G/C]	Sp1	Transfac:v7.0:M00008	ccGG[g/C]GCggg	1.000/0.949	-	pMATCH	(y|N)
					19:47056289-47056298:+1			(minSUM)	
			Sp1	Transfac:v7.0:M00008	cgG[G/c]GCGggc	-	0.957/0.956		(y|N)
					19:47056290-47056299:+1				
			Sp1	Transfac:v7.0:M00008	gg[G/c]GCGGgcc	-	1.000/0.985		(y+|N)
					19:47056291-47056300:+1				
			Pax-2	Transfac:v7.0:M00098	gg[g/c]gCGGGCccgtcccgcc	0.938/0.891	-		(y+|N)
					19:47056291-47056309:+1				
			Pax-5	Transfac:v7.0:M00144	cccgaGgcgcccgg[g/c]GCGggcCcgtccc	84.2745/0.9	379.43/0.9	MotifScanner	(y|N)
					19:47056279-47056306:+1				
			Pax-5	Transfac:v12.1:M00144	cccgaggcgcccgG[G/C]GCGggcccgtccc	0.839/0.753	0.873/0.767	MATCH	(y|N)
					19:47056279-47056306:+1			(minFP)	
**upstream**	**novel-13**	[A/G]	PU.1 (Spi-1)	Jaspar:v7.0:MA0080	cGGA[A/g]c	-/0.9	311.828/0.9	MotifLocator	(Y|N)
					19:47056538-47056543:+1				
			Pax-2	Transfac:v7.0:M00098	ggagCCGGA[a/g]cccggcgtt	-	0.969/0.893	pMATCH	(y|N)
					19:47056533-47056551:+1			(minSUM)	
**upstream**	**novel-14**	[A/G]	Pax-2	Transfac:v7.0:M00098	cggcGTTGAagg[a/g]gccgtg	0.982/0.935	0.982/0.939	pMATCH	(y|N)
					19:47056545-47056563:+1			(minSUM)	
			Pax-2	Transfac:v7.0:M00098	ggcgTTGAAgg[a/g]gccgtgg	0.955/0.930	0.955/0.941		(y|N)
					19:47056546-47056564:+1				
			Msx-1	Transfac:v7.0:M00394	cgtTGAAGg[a/g]	0.963/0.938	0.963/0.938		(y−|N)
					19:47056548-47056556:+1				
			HOXA3	Transfac:v7.0:M00395	CGTTGaagg[a/g]	1.000/0.976	1.000/0.976		(y−|N)
					19:47056548-47056556:+1				
			HOXA3	Transfac:v7.0:M00395	TTGAAgg[a/g]g	-	0.973/0.943		(y−|N)
					19:47056550-47056558:+1				
			HOXA3	Transfac:v7.0:M00395	TGAAGg[a/g]gc	1.000/0.954	1.000/0.954		(y−|N)
					19:47056551-47056559:+1				
			Sp1	Transfac:v7.0:M00008	gg[a/G]GCCGtgg	0.981/0.942	-		(y+|N)
					19:47056555-47056564:+1				
**upstream**	**novel-15**	[A/G]	Pax-2	Transfac:v7.0:M00098	ggggGTACCac[a/g]gtttagg	0.895/0.891	-	pMATCH	(y|N)
					19:47056574-47056592:+1			(minSUM)	
			Pbx-1	Transfac:v7.0:M00096	accAC[A/g]GTt	-	0.930/0.937		(y+|N)
					19:47056580-47056588:+1				
			HOXA3	Transfac:v7.0:M00395	C[A/G]GTTtagg	0.956/0.953	0.940/0.941		(y−|N)
					19:47056584-47056592:+1				
			Msx-1	Transfac:v7.0:M00394	c[a/g]gTTTAGg	0.940/0.959	0.940/0.959		(y−|N)
					19:47056584-47056592:+1				
			HOXA3	Transfac:v7.0:M00395	ACCAC[a/g]gtt	1.000/0.995	1.000/0.995	pMATCH	(y+|N)
					19:47056580-47056588:+1			(minSUM, minFP)	
			Pax-2	Jaspar:v3.0:MA0067	tacCAc[a/g]g	112.092/0.9	/0.9	MotifLocator	(Y|N)
					19:47056579-47056586:+1				
**2^nd^ intron**	**rs2075749**	[C/G]	Sp1	Transfac:v7.0:M00008	[c/g]GAGGCTTGTT	111.864/0.3	188.282/0.3	MotifScanner	(y+|N)
					19:47056845-47056854:+1				
**4^th^ intron**	**rs1366610**	[A/G]	Mzf1	Transfac:v7.0:M00084	agggtAG[a/G]GGggg	201.027/0.5	-/0.5	MotifScanner	(y|N)
					19:47065131-47065143:+1				
			MZF1_5-13	Jaspar:v3.0:MA0057	gtAg[a/G]GGggg	1253.7/0.5	-/0.5		(y|N)
					19:47065134-47065143:+1				
			Sp1	Jaspar:v3.0:MA0079	ag[a/G]Gggggct	104.692/0.5	-/0.5		(y|N)
					19:47065136-47065145:+1				
**4^th^ intron**	**novel-41**	[C/G]	Mzf1	Transfac:v7.0:M00084	agggtAGGGGg[c/g]g	201.027/0.5 -/0.5	-/0.5	MotifScanner	(y|N)
					19:47065131-47065143:+1				
			MZF1_5-13	Jaspar:v3.0:MA0057	gtAgGGGg[c/g]g	1253.7/0.5	-/0.5		(y−|N)
					19:47065134,47065143:+1				
			SP1	Jaspar:v3.0:MA0079	agGGgg[c/g]gct	104.692/0.5	-/0.5		(y−|N)
					19:47065136-47065145:+1				
**4^th^ intron**	**novel-42**	[T/G]	HOXA3	Transfac:v7.0:M00395	ac[T/g]Aatggt	-/0.4	121.962/0.4	MotifScanner	(y|N)
					19:47065188-47065196:+1				

# For a complete list of identified TFBS coinciding with detected SNPs see supplementary table S1.

a location of the SNP/TFBS with respect to the *RPS19* open reading frame.

b database identifier (dbSNP) for previously described SNPs or number of novel SNP, respectively.

c SNP alleles, indicating reference and alternative (replace) allele.

d name of transcription factor or motif recognized, as named in the databases.

e library, version of the library and identifier to which a TFBS is associated under a PWM database release.

f motif recognized (5′ to 3′ sequence). Allele positioning marked in bold. Alleles falling exactly adjacent to the end of a motif highlighted in bold and italics. Capital letters highlight important positions for the putative binding strength of a motif. Chromosome, start, end position and strand within the human reference genome (hg18, build 36.1).

g score for the reference genome allele. MATCH and pMATCH use 1.000 as maximum score between an optimal binding site match and matrix power of detection. TOUCAN detection tools (MotifScanner, MotifLocator, MotifSampler) do not use global maximum or matrix scoring, but the higher the numbers, the better the predicted site. The *apriori* probability or threshold is stated under the score when any of the TOUCAN tools has been used.

h score for non-reference allele.

i program used for detection of a motif. Error minimization criteria stated when applicable.

j transcript factor contained in any of the multi-conserved sequence (MCS) region of the multi-species alignment. The first value belongs to the Infocon program and the second to the EPO EnsEMBL track. ‘Y’ indicates that the TFBS motif is totally contained in the area (see supplementary figure S1 and file S2), ‘y+’ that there is major overlapping part (>75%), ‘y’ that there is significant overlapping part (<75%, >50%), ‘y−’ only if a minor part overlaps (<50%) and ‘N’ indicates an inexistent overlap.

### Comparative analysis of the proximal promoter region of *RPS19*

Previous studies have tried to identify the promoter region of *RPS19*. Our study confirms that the *RPS19* promoter region shares typical features with other mammalian ribosomal protein genes (e.g. absence of a canonical TATA-box). We also detected an accompanying non-consensus CCATT-box 72 to 83 bp upstream of the transcription start site commonly described in public databases (according to EnsEMBL). Three different transcription start sites (TSS) have been described (BC018616; BC000023; D28389) which differ only in the length of the 5′UTR of the resulting mRNA. We have observed additional 5′UTR variants differing in length between 33 to 467 nucleotides (unpublished data). The observed spread of the TSS together with the absence of a canonical TATA-box classify the *RPS19* promoter as “broad type promoter” according to Sandelin and colleagues [25]. Interestingly, the TSS stretch encompasses regions important for expression of *RPS19* described previously by DaCosta et al. (figure 1) [26]. The authors identified regions of high conservation between mouse and human in the putative promoter region of *RPS19*, regions we also detect in our analysis (figure 1A). They predicted a promoter sequence and subsequently showed that the predicted promoter sequence and one of the conserved regions are important for expression of a reporter construct [26].

We did not detect any variation in the 1.5 kb proximal region upstream of the first exon. This region is additionally characterized by a high degree of conservation (figure 1_1). These findings underscore the importance of this region for expression of *RPS19*. DaCosta and colleagues identified several putative TFBS within the upstream region of *RPS19* [26]. We checked whether we could reproduce their predictions of TFBS. In several cases, we obtained an even finer consensus motif (e.g. n-Myc, figure 1). In their study, DaCosta and coworkers define a strong C-Rel/Rel-A site that coincides with an NF-κB site. This is not surprising, because NF-κB is known to bind C-Rel in transcriptional regulatory systems [27]. Our matrix used in this detection is actually capable of detecting such a consensus site but cataloged it as NF-κB. Finally, we detected the SP1 motif in the first conserved region and an SP1 instead of the CACCC-Bf binding site [26]. This particular SP1 binding site (CCACCC) has been described as a regulatory switch element that stimulates SP1/GATA1 cooperation, and the consensus sequence is similar to the CACCC-Bf sequence [28].

## Discussion

Association studies take advantage of the known variations throughout the human genome including SNPs and microsatellites. It has been suggested that the identification of all the potential risk-conferring variations within one disease associated gene is important for appropriate genotype-phenotype correlations [29], [30]. Targeted resequencing studies are therefore an important step that may provide detailed catalogs of genomic variations to further studies of the mechanisms underlying diseases and pharmacogenetic responses [31]. We focused our efforts on the resequencing analysis of the *RPS19* gene locus, a region that has been linked to two forms of anemia, namely Diamond-Blackfan Anemia (DBA) and Transient Erythroblastopenia of childhood (TEC) [12], [32]. Our initial goal was to identify non-coding polymorphisms that are associated with either disease.

In the present resequencing study, we show a considerable and previously unrecognized variation within the *RPS19* gene locus. Estimates have approximated the degree of variation for chromosome 19 to 1 SNP in about 2 kbp of sequence [19], [21]. We show here that the genetic variation at the *RPS19* locus in our patient cohort is significantly higher, with 1 SNP per 294 bp, and provide a catalog of additional variations associated with DBA and TEC. A large proportion of the presented variations consists of “private” SNPs. Interestingly, independent resequencing studies of e.g. the innate immunity genes and the APO gene cluster have also detected an unexpectedly high degree of variation [20], [29]. The high degree of variation in this study and the fact that *RPS19* seems to play a central role in a large proportion of DBA patients suggest that regulatory networks altered by one or the other SNP may have implications for *RPS19* expression.

However, our results revealed no clear correlation between any of the identified SNPs and either of the DBA or TEC phenotypes. Linkage analyses have previously indicated co-segregation of the two disorders suggesting they are allelic variants [4], [12], [32]. This lack of phenotype-genotype correlation may indicate that there exists an as yet unidentified sequence element in this region responsible for the regulation of *RPS19* expression. Indeed, it has been described that regulatory elements may be situated far away from the actual gene. Mutations in such elements have previously been implicated in human diseases [33]. Alternatively, the observed linkage of this region to TEC patients is not due to mutations in *RPS19*, but to a different gene within the 1 Mbp region described previously [4]. Although the region contains a number of genes, no other ribosomal protein gene is located within this 19q13.2 region and no candidate gene of known relevance for erythropoiesis could be identified.

Consequently, we hypothesized that mutations in non-coding regions of *RPS19* could disrupt the binding of regulatory proteins. We aimed to identify new regulatory modulators and carried out a bioinformatics analysis of the locus to identify putative transcription factor binding sites (TFBS). As a result, we obtained a catalog of variations within our patient cohort and we provide a map of putative transcription factor binding sites (table 2 and supplementary table S1). Several of the corresponding transcription factors (TF) are of particular interest. Ten of the identified TFs are ubiquitously or widely expressed (i.e. general transcription factors) and important for regulation of development, cell cycle and cell division, and cell plasticity (Cell division control protein 5 (CDC5); Homeobox cluster protein A3 (HOXA3); Msh-like homebox protein 1 (MSX-1); Paired box transcription factors (PAX); Peroxisome proliferator-activated receptor (PPARalpha and gamma); SP1 transcription factor (SP1); YY1 transcription factor (YY1)) [34]–[36]. Variations in the TFBS of these factors could possibly lead to altered transcriptional activity of the *RPS19* gene, as has been described previously for transcription factors and the Ebox module [37]–[40]. A marked reduction in the transcriptional activity of *RPS19* may have effects similar to that observed for haploinsufficiency. Strikingly, the non-reference allele of one SNP (rs3214574) deletes the putative binding site for CDC5. Instead, a strong TFBS for GATA-1/2 is created. This suggests a significant change for tissue specific expression. Moreover, the general TFs as well as the Ebox binding site could be pivotal for cellular response to extracellular stimuli and this may explain individual response to treatment or endogenous cytokines [41]–[43]. Furthermore, several of these general transcription factors have been shown to play a role in cell proliferation and tumorigenesis for which ribosomal protein genes are essential [44]–[47].

Six TFBS identified in our study are even more interesting (GATA binding proteins 1 and 2 (GATA-1 and GATA-2); Myeloid zinc finger 1 (MZF-1); Pre-B-cell leukemia homeobox 1 (PBX-1); hematopoietic transcription factor PU.1 (PU.1); Ebox binding site). They belong to factors with a direct link to hematopoiesis [48]–[53]. Several of these factors are involved in the progression to leukemia and they are essential for normal hematopoiesis (e.g. PU.1). We speculate that these factors may play a crucial role in the transcription of *RPS19* during hematopoiesis, and alterations in the respective TFBS could lead to diminished *RPS19* expression. This might render erythroid precursors to be less capable to proliferate, which has been suggested as a mechanism underlying DBA in patients with mutations in the coding sequence of *RPS19* [14]. On the other hand, alterations in TFBS could also lead to increased levels of RPS19 and in the best case promote remission which is seen to occur spontaneously.

Another possible mechanism is that specific alleles in SNPs overlapping with the TFBS for PU.1, GATA-1/2 or PBX-1 might be of importance for the development of hematopoietic stem cells by altering their capacity of self-renewal, expansion and quiescence [50], [53], [54]. These factors are candidates in the mechanism underlying a block in erythroblast expansion and differentiation in DBA patients [55].

In summary, we report here on the considerable individual variation detected in our resequencing study of the disease locus *RPS19* in DBA and TEC patients. Furthermore, we identified a series of transcription factors putatively involved in the regulation of *RPS19* expression and implicated in the pathobiology of DBA and TEC. Functional follow-up studies are needed to further investigate the predicted interactions described in this report.

## Methods

### Ethics Statement

The study was approved by the Regional Ethical Review Board of Uppsala (Diary Number 2006/118). Informed consent of patients or their parents was obtained and has been documented in the patient files by the responsible clinician following routines approved by the Regional Ethics Board and according to Swedish legislation.

### Patient cohort

We analyzed DNA prepared from peripheral blood of 77 DBA and 12 TEC patients of Caucasian origin. Patients included in the study were excluded by sequencing to carry a structural mutation in *RPS17*, *RPS19* or *RPS24*, respectively (Primer sequences available on request). Most of the patients are sporadic cases, except for 10 of the DBA patients and eight of the TEC patients who previously showed association with this genomic region. All patients were ascertained by hematologists of their country for criteria for DBA or TEC, respectively, and have been described previously [4], [12], [32].

### Resequencing

Resequencing was carried out as described (METHOD-A; dbSNP (http://www.ncbi.nlm.nih.gov/SNP/)). Additional sequence analysis was performed by sequencing standard PCR products (from approximately 2 µg genomic DNA) in both directions on an ABI PRISM® 3700 DNA Analyzer (AppliedBiosystems) according to manufacturer's protocol and using Sequencher® Programme for analysis of the resulting sequences. Primer sequences are listed in supplementary table S2.

### Comparative genomic sequence analysis

200 kb of the human genome sequence around the *RPS19* gene locus (hg18; chr19:47,048,239–47,068,000) as well as orthologous sequences for six mammalian species in different orders (rodents [mouse], canines [dog], ungulates [cow], primates [orangutan, macaque and chimpanzee]) were retrieved from EnsEMBL (http://www.ensembl.org/Homo_sapiens/) [56], assuring gene synteny was conserved and gaps were not extensive (supplementary text S1 and supplementary figure S1). MultiPipMaker aligned these orthologous sequences with the ‘single coverage’ option to eliminate matches caused by duplications and the ‘search both strand’ option [57]. The identified multi-species conserved sequences were analyzed by virtue of the Infocon program [24]. Infocon identifies blocks of high information content (BHIC) in parts of the alignment and optionally calculates a consensus sequence in each block. A BHIC is a cluster of conservation between species in which the alignment contains information for every species considered for the alignment.

In order to obtain a more informative alignment of the mammalian clade, the multi-species alignment EPO track was downloaded from EnsEMBL containing elements conserved along 29 eutherian mammals and subsequently converted into a *.gff file (supplementary texts S1 and S2). For a description of the *.gff file format see http://www.sanger.ac.uk/Software/formats/GFF/GFF_Spec.shtml.

### Prediction of transcription factor binding motifs

Sequences of the whole human *RPS19* upstream gene region (hg18; chr19:47,048,239–47,056,684) as well as 2^nd^ and 4^th^ introns (chr19:47,056,756–47,057,020 and chr19:47,065,125–47,065,608, respectively) and the 6 mammalian species already mentioned were subjected to a transcription factor binding sites (TFBS) detection with help of various programs: MotifScanner, MotifLocator, MotifSampler, MATCH and pMATCH.

These programs identify over-represented motifs in a sequence data set and annotate putative binding sites consulting libraries of position weight matrices (PWMs). PWMs represent the intrinsic sequence variability of TFBS in the form of a matrix. Each matrix stores the frequency for each nucleotide at every position of the putative motif in order to summarize the alignment information for the TF with a binding site. The libraries of PWMs used in this study were Jaspar [58] and TRANSFAC Professional (releases 7.0 (public), 11.2 and 12.1) [59]. The professional version of TRANSFAC requires licensing.

MotifScanner uses different orders of markov chains as background model for matching PWMs. A parameter called a ‘prior’ assigns an a priori probability of TFs binding to a distinct sequence. In MotifLocator the ‘prior’ is substituted by a posterior threshold for filtering matching PWMs. The resulting scores, in an absolute scale without log correction, represent the likelihood ratio of a certain PWM match versus a random match. MotifSampler [60] implements a stochastic model of a Gibbs sampler to detect “over-represented” motifs not matching any known PWM. We always used the default parameters and third order vertebrate and human models (Eukaryotic Promoter Database and dbTSS) for MotifScanner and MotifLocator when searching against TRANSFAC and Jaspar libraries. All these programs are contained in a workbench for regulatory sequence analysis called TOUCAN [61].

MATCH [62] and pMATCH [63] are closely interconnected with the TRANSFAC database. MATCH was executed to minimize the false positives error (minFP) to guarantee specificity, whilst pMATCH was selected with minimization for FP and combined with the sum of both false positive and false negative errors (minSUM) to increase sensitivity.

There are other recognition tools for promoter and regulatory motif analysis available: RSAT (Universite Libre de Bruxelles), TESS (University of Pennsylvania), TSSG/TSSW (Baylor College of Medicine), MatInspector (Genomatix Gmbh), SiteSeer (University of Manchester), AliBaba2 (BioBase Gmbh), FUNSITE (ICG), Footprinter (University of Washington). However, we used the tools best integrated with the libraries used in the study.

Additionally, several Perl scripts were created to perform data analysis in the suitable tools, parse TFBS annotations into *.gff files and filter overlapping SNPs within selected TFBS. All available annotations of the locus were manually formatted into *.gff files (supplementary text S2). Images of the *RPS19* locus containing variations, conserved areas, annotations and TFBS provided within this report were created using the UCSC Genome Browser (http://www.genome.ucsc.edu/) [64].

## Supporting Information

Figure S1 Detailed view of the analyzed region. Every *.gff file available has been imported into the UCSC Genome Browser (Kuhn et al, 2009) for visualisation (see supplementary text S2).
(1.71 MB PDF)

Figure S2 Gene structures of the orthologous RPS19 loci of the species selected for comparative analysis taken from EnsEMBL (Hubbard et al, 2009).
(1.09 MB PDF)

Table S1 Full list of all detected TFBS overlaying identified variations. a location of the SNP/TFBS with respect to the RPS19 open reading frame b database identifier (dbSNP) for previously described SNPs or number of novel SNP, respectively. We complement this field with a small registry of which TFBSs are created (+) or destroyed (−) in a certain SNP (the actual SNP if not stated) and the detection score for this binding site if applicable. c SNP alleles, indicating reference and alternative (replace) allele d name of transcription factor or motif recognized, as named in the databases e library, version of the library and identifier to which a TFBS is associated under a PWM database release f motif recognized (5′ to 3′ sequence). Allele positioning marked in bold. Alleles falling exactly adjacent to the end of a motif highlighted in bold and italics. Capital letters highlight important positions for the putative binding strength of a motif. Chromosome, start, end position and strand within the human reference genome (hg18, build 36.1) g score for the reference genome allele. MATCH and pMATCH use 1.000 as maximum score between an optimal binding site match and matrix power of detection. TOUCAN detection tools (MotifScanner, MotifLocator, MotifSampler) do not use global maximum or matrix scoring, but the higher the numbers, the better the predicted site. The a priori probability or threshold is stated under the score when any of the TOUCAN tools has been used h score for non-reference allele i program used for detection of a motif. Error minimization criteria stated when applicable j transcript factor contained in any of the multi-conserved sequence (MCS) region of the multi-species alignment. The first value belongs to the Infocon program and the second to the EPO EnsEMBL track. ‘Y’ indicates that the TFBS motif is totally contained in the area (see supplementary figure S1 and text S2), ‘y+’ that there is major overlapping part (>75%), ‘y’ that there is significant overlapping part (<75%, >50%), ‘y−’ only if a minor part overlaps (<50%) and ‘N’ indicates an inexistent overlap
(0.16 MB PDF)

Table S2 Primer sequences for analysis of 5′upstream region.
(0.16 MB PDF)

Text S1 Sequences collected for the bioinformatic analyses (in fasta format).
(0.07 MB TXT)

Text S2 Compressed archive with files used in the study for visualization in the UCSC Genome Browser (in *.gff format)
(0.16 MB ZIP)
