# Supplementary figures and images for: Targeted Resequencing and Analysis of the Diamond-Blackfan Anemia Disease Locus RPS19
Source: PLoS One. 2009 Jul 9;4(7):e6172. doi: 10.1371/journal.pone.0006172 (PMC2703794; doi:10.1371/journal.pone.0006172)

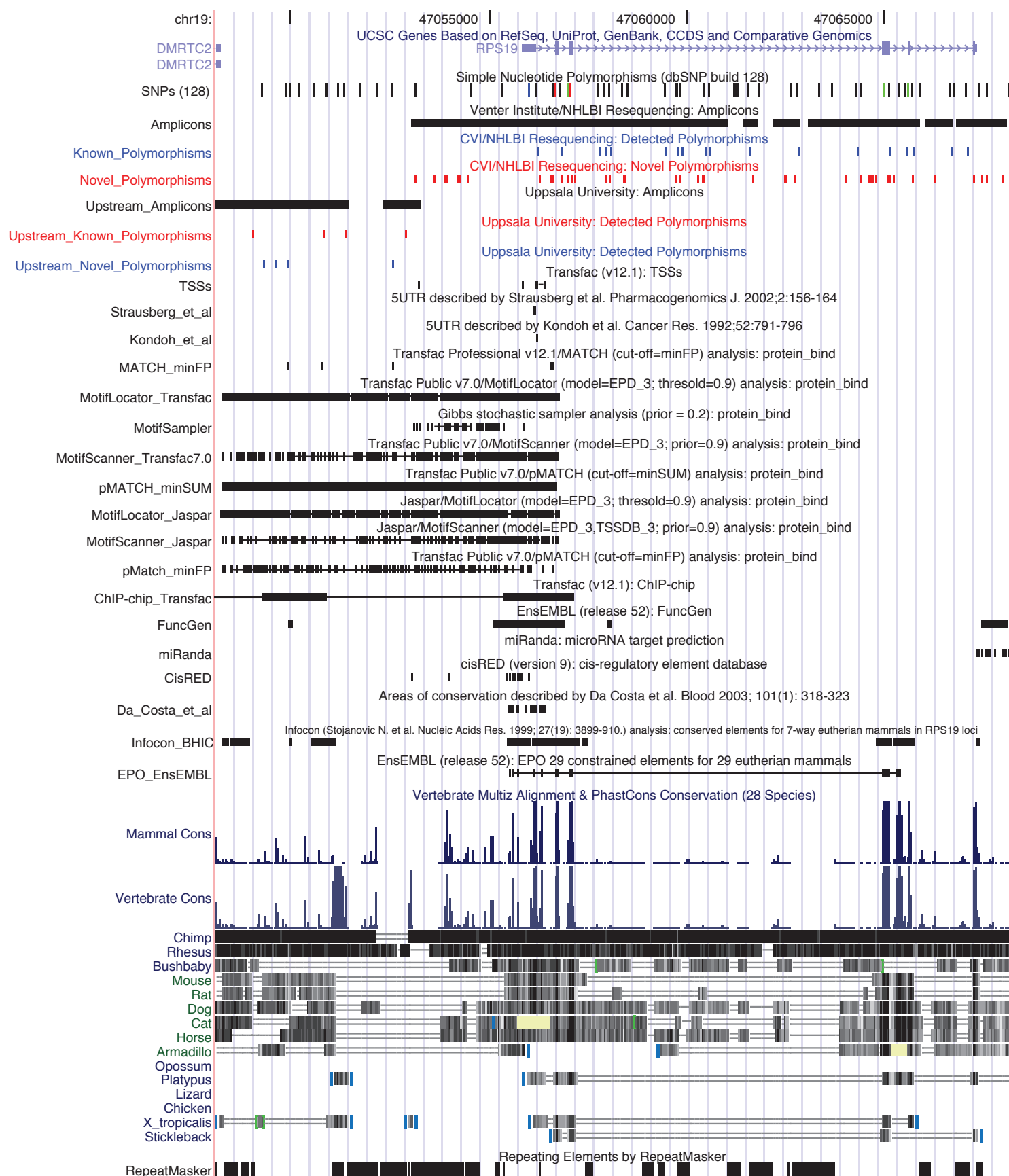

Barrio\_Supplementary Figure S2

Supplement: Figure S2 — Gene structures of the orthologous RPS19 loci of the species selected for comparative analysis taken from EnsEMBL (Hubbard et al, 2009). (1.09 MB PDF) [file pone.0006172.s002.pdf]
