# Supplementary material for: Targeted Resequencing and Analysis of the Diamond-Blackfan Anemia Disease Locus RPS19
Source: PLoS One. 2009 Jul 9;4(7):e6172. doi: 10.1371/journal.pone.0006172 (PMC2703794; doi:10.1371/journal.pone.0006172)
Supplement: Table S1 — Full list of all detected TFBS overlaying identified variations. a location of the SNP/TFBS with respect to the RPS19 open reading frame b database identifier (dbSNP) for previously described SNPs or number of novel SNP, respectively. We complement this field with a small registry of which TFBSs are created (+) or destroyed (−) in a certain SNP (the actual SNP if not stated) and the detection score for this binding site if applicable. c SNP alleles, indicating reference and alternative (replace) allele d name of transcription factor or motif recognized, as named in the databases e library, version of the library and identifier to which a TFBS is associated under a PWM database release f motif recognized (5′ to 3′ sequence). Allele positioning marked in bold. Alleles falling exactly adjacent to the end of a motif highlighted in bold and italics. Capital letters highlight important positions for the putative binding strength of a motif. Chromosome, start, end position and strand within the human reference genome (hg18, build 36.1) g score for the reference genome allele. MATCH and pMATCH use 1.000 as maximum score between an optimal binding site match and matrix power of detection. TOUCAN detection tools (MotifScanner, MotifLocator, MotifSampler) do not use global maximum or matrix scoring, but the higher the numbers, the better the predicted site. The a priori probability or threshold is stated under the score when any of the TOUCAN tools has been used h score for non-reference allele i program used for detection of a motif. Error minimization criteria stated when applicable j transcript factor contained in any of the multi-conserved sequence (MCS) region of the multi-species alignment. The first value belongs to the Infocon program and the second to the EPO EnsEMBL track. ‘Y’ indicates that the TFBS motif is totally contained in the area (see supplementary figure S1 and text S2), ‘y+’ that there is major overlapping part (>75%), ‘y’ that there is significant overlap [file pone.0006172.s003.pdf]

Region [a]	SNP [b]	RefSNP [c]	TFBS name [d]	Source [e]	Motif [f]<br>Position(chr:start-end:strand)	Ref allele score [g]	Non-ref allele score [h]	Tool [i]	MCS [j]
**upstream**	**novel-1**<br>(+YY1)	[G/A]	YY1 (Yin and Yang 1)	Transfac:v7.0:M00059	taatCCCAGc[G/a]ctttgg<br>19:47049005-47049021:+1	-	0.996/0.976	pMATCH (minSUM)	(N | N)
			Pax-2 (Paired box 2)	Transfac:v7.0:M00098	aatcCCAGC[g/a]ctttgggag<br>19:47049006-47049024:+1	0.992/0.943	-		(N | N)
			Pax-2	Transfac:v7.0:M00098	atccCAGC[G/A]ctttgggagg<br>19:47049007-47049025:+1	0.918/0.940	0.910/0.936		(N | N)
			HOXA3 (Homeobox cluster protein)	Transfac:v7.0:M00395	AGC[G/A]Ctttg<br>19:47049012-47049020:+1	1.000/0.963	-		(N | N)
			HOXA3	Transfac:v7.0:M00395	C[G/A]CTTtggg<br>19:47049014-47049022:+1	0.940/0.941	0.956/0.953		(N | N)
			CdxA (CdxA homeobox gene)	Transfac:v7.0:M00100	[g/a]CTTTGgg<br>19:47049016-47049022:+1	1.000/0.992	1.000/0.992		(N | N)
**upstream**	**rs4803512**<br>(+HOXA3)<br>(- 2 x Pax-2, YY1, 2 x HOXA3)	[A/C]	YY1	Transfac:v7.0:M00069	ttgaACCATtc[a/c]gtaca<br>19:47049284-47049300:+1	0.996/0.982	-	pMATCH (minSUM)	(N | N)
			Pax-2	Transfac:v7.0:M00098	tgaaCCATTc[a/c]gtacatag<br>19:47049285-47049303:+1	0.982/0.922	0.982/0.919		(N | N)
			Evi-1 (Ectopic viral integration site 1)	Transfac:v7.0:M00081	aaccattc[a/c]GTACAt<br>19:47049287-47049301:+1	0.794/0.863	0.794/0.863		(N | N)
			HOXA3	Transfac:v7.0:M00395	ACCATtc[a/c]g<br>19:47049288-47049296:+1	0.954/0.948	0.954/0.948		(N | N)
			Pax-2	Transfac:v7.0:M00098	accaTTC[A/C]Gtacataggaa<br>19:47049288-47049306:+1	0.947/0.891	-		(N | N)
			Pax-2	Transfac:v7.0:M00098	ccatTC[A/C]GTacataggaaa<br>19:47049289-47049307:+1	0.975/0.903	-		(N | N)
			CdxA	Transfac:v7.0:M00100	CATTC[a/c]g<br>19:47049290-47049296:+1	1.000/0.975	1.000/0.971		(N | N)
			HOXA3	Transfac:v7.0:M00395	CATTC[a/c]gta<br>19:47049290-47049298:+1	0.970/0.946	-		(N | N)
			HOXA3	Transfac:v7.0:M00395	TTC[A/C]Gtaca<br>19:47049292-47049300:+1	1.000/0.975	-		(N | N)
			Pax-2	Transfac:v7.0:M00098	ttc[a/c]GTACAtaggaaaacc<br>19:47049292-47049310:+1	0.981/0.946	0.981/0.945		(N | N)
			HOXA3	Transfac:v7.0:M00395	[A/C]GTACatag<br>19:47049295-47049303:+1	-	0.957/0.961		(N | N)
			Msx-1 (Msh-like homeobox protein 1)	Transfac:v7.0:M00394	c[a/c]gTACATa<br>19:47049294-47049302:+1	1.000/1.000	1.000/1.000	pMATCH (minSUM, minFP)	(N | N)
**upstream**	**rs3214574**<br>(+Pax-6, C/EBPalpha, C/EBPbeta, 2 x Evi-1, C/EBP)<br>(- HOXA3, 2 x Evi-1)<br>(+ Pax-2, novel-6;)<br>(- Spz1, novel-15;)	[-/CT]	Pax-2	Transfac:v7.0:M00098	cttcCCTCCccttag[-/ct]at<br>19:47049605-47049623:+1	0.924/0.893	0.924/0.894<br>(cttcCCTCCccttagataa)	pMATCH (minSUM)	(N | N)
			HOXA3	Transfac:v7.0:M00395	CCCCTtag[-/ct]<br>19:47049612-47049620:+1	0.926/0.942	-		(N | N)
			C/EBPalpha<br>(CCAAT/enhancer binding protein)	Transfac:v7.0:M00116	ccCTTAGataactt<br>19:47049613-47049626:+1	-	1.000/0.997		(N | N)
			C/EBPbeta	Transfac:v7.0:M00117	cccTTAGAtaactt<br>19:47049613-47049626:+1	-	1.000/0.977		(N | N)
			HOXA3	Transfac:v7.0:M00395	CCTTAg[-/ct]a<br>19:47049614-47049622:+1	0.987/0.942	0.987/0.959<br>(CCTTAgata)		(N | N)
			Evi-1	Transfac:v7.0:M00081	ccttagataACTTAg<br>19:47049614-47049628:+1	-	0.805/0.874		(N | N)
			C/EBP	Transfac:v7.0:M00159	ccTTAGAtaactt<br>19:47049614-47049626:+1	-	0.980/0.973		(N | N)
			Msx-1	Transfac:v7.0:M00394	cctTAG[-/CT]a<br>19:47049614-47049622:+1	0.955/0.933	0.962/0.938<br>(cctTAGATa)		(N | N)
			Evi-1	Transfac:v7.0:M00081	ccttagataACTTAg<br>19:47049614-47049628:+1	-	0.805/0.874		(N | N)
			HOXA3	Transfac:v7.0:M00395	CTTAG[-/ct]at<br>19:47049615-47049623:+1	0.957/0.943	-		(N | N)
			Evi-1	Transfac:v7.0:M00081	ag[-/ct]ataacTTAGAt<br>19:47049618-47049632:+1	0.898/0.844	-		(N | N)
			Pax-2	Transfac:v7.0:M00098	ag[-/ct]ATAACttagatatat<br>19:47049618-47049636:+1	0.972/0.897	0.972/0.896<br>(ttagATAACttagatatat, 19:47049616-47049634:+1)		(N | N)
			Oct-1 (octamer-binding factor 1)	Transfac:v7.0:M00137	g[-/ct]ATAACttaga<br>19:47049619-47049631:+1	0.996/0.992	0.996/0.992<br>(tagATAACttaga, 19:47049617-47049629:+1)		(N | N)
			Evi-1	Transfac:v7.0:M00081	[-/ct]ataacttAGATAt<br>19:47049620-47049634:+1	0.931/0.828	-		(N | N)
			Oct-1	Transfac:v7.0:M00162	[-/ct]ataacttaGATATa<br>19:47049622-47049635:+1	0.982/0.960	0.982/0.960<br>(19:47049620-47049633:+1)		(N | N)
			Pax-6	Transfac:v7.0:M00097	cctccCCTTAgataacttaga<br>19:47049609-47049629:+1	-	0.987/0.976	pMATCH (minSUM, minFP)	(N | N)
			HOXA3	Transfac:v7.0:M00395	[-/ct]ATAACttag<br>19:47049622-47049630:+1	1.000/0.995	1.000/0.995		(N | N)
			GATA-1 (GATA-binding factor 1)	Transfac:v7.0:M00346	ttaGATAAct<br>19:47049616-47049625:+1	-/0.9	1629.46/0.9	MotifScanner	(N | N)
			GATA-2 (GATA-binding factor 2)	Transfac:v7.0:M00082	ttaGATAAct<br>19:47049616-47049625:+1	-/0.9	3124.5/0.9		(N | N)
			Evi-1	Transfac:v7.0:M00082	AGATAACTTAG<br>19:47049618-47049628:+1	-/0.9	670.903/0.9		(N | N)
			Cdc5 (Cdc5 cell division control protein 5)	Transfac:v7.0:M00478	G[-/ct]aTAacttag<br>19:47049619-47049630:+1	3343.59/0.9	-/0.9		(N | N)
**upstream**	**rs6509002**<br>(- Evi-1, RORalpha1, Pax-2, PPARalpha, PPARgamma)	[C/T]	Pax-2	Transfac:v7.0:M00098	aggaGTAGActagagg[c/t]ca<br>19:47049893-47049911:+1	0.915/0.916	0.915/0.916	pMATCH (minSUM)	(y+ | N)
			Evi-1	Transfac:v7.0:M00081	gagtagactAGAGG[c/t]<br>19:47049895-47049909:+1	0.773/0.866	0.773/0.866		(y+ | N)
			Pax-2	Transfac:v7.0:M00098	agtaGACTAgagg[c/t]cacct<br>19:47049896-47049914:+1	0.840/0.906	0.840/0.906		(y | N)
			Evi-1	Transfac:v7.0:M00081	gactagagg[c/T]CACCt<br>19:47049900-47049914:+1	0.796/0.885	-		(y+ | N)
			RORalpha1<br>(RAR-related orphan receptor alpha1)	Transfac:v7.0:M00156	gactagaGG[c/T]CAc<br>19:47049900-47049912:+1	1.000/0.994	-		(y+ | N)
			Pax-2	Transfac:v7.0:M00098	agagG[c/T]CACctctccttca<br>19:47049904-47049922:+1	0.992/0.914	-		(y- | N)
			Evi-1	Transfac:v7.0:M00082	gactAGAGG[c/t]c<br>19:47049900-47049910:+1	0.975/0.987	0.975/0.981	pMATCH (minSUM, minFP)	(y+ | N)
			PPARalpha (PPAR:RXR heterodimers)	Transfac:v12.1:M00242	aAggAgTAGactAgAGG[c/T]CA<br>19:47049892-47049911:+1	0.784/0.784	-	MATCH (minFP)	(y+ | N)
			PPARgamma	Transfac:v7.0:M00528	gagtaGactAgaGg[c/T]ca<br>19:47049895-47049911:+1	3331.06/0.9	-/0.9	MotifScanner	(y | N)
**upstream**	**novel-3**<br>(+ Pax-2, COMP-1)	[C/T]	Pax-2	Transfac:v7.0:M00098	cagaGTTCCcta[c/t]gttccc<br>19:47050781-47050799:+1	-	0.915/0.916	pMATCH (minSUM)	(Y | N)
			Pax-2	Transfac:v7.0:M00098	agagTTCCCta[c/t]gttccca<br>19:47050782-47050800:+1	0.982/0.911	0.982/0.899		(Y | N)
			C/EBPalpha	Transfac:v7.0:M00116	gaGTTCCcta[c/t]gtt<br>19:47050783-47050796:+1	0.999/0.995	0.999/0.996		(y | N)
			COMP1	Transfac:v7.0:M00057	agttccCTA[C/t]Gttcccaaggggcc<br>19:47050784-47050807:+1	-	0.878/0.833		(y- | N)
			Msx-1	Transfac:v7.0:M00394	cccTA[C/T]GTt<br>19:47050788-47050796:+1	0.955/0.940	0.992/0.965		(Y | N)
			HOXA3	Transfac:v7.0:M00395	CCTA[C/T]gttc<br>19:47050789-47050797:+1	0.987/0.943	1.000/0.945		(Y | N)
			Pax-2	Transfac:v7.0:M00098	cta[c/t]GTTCCcaaggggcca<br>19:47050790-47050808:+1	0.963/0.928	0.963/0.928		(y+ | N)
			PPARgamma2<br>(peroxisome proliferator-activated receptor gamma)	Transfac:v12.1:M00515	ctcTcaGgCAgaGTtcCCtA[C/t]gT<br>19:47050773-47050795:+1	0.604/0.675	0.604/0.682	MATCH (minFP)	(y+ | N)
			box_1_2_CTATGT	Model:EPD Human 3*	CTA[c/T]GT<br>19:47050790-47050795:+1	6336.9	N.A.	MotifSampler	(Y | N)
**upstream**	**rs7258162**<br>(- Msx-1, HOXA3)	[C/G]	Pax-2	Transfac:v7.0:M00098	gcgcCCACCacct[c/g]cccca<br>19:47052562-47052580:+1	0.992/0.947	0.992/0.921	pMATCH (minSUM)	(N | N)
			Msx-1	Transfac:v7.0:M00394	cacCACCT[c/g]<br>19:47052567-47052575:+1	0.906/0.936	-		(N | N)
			HOXA3	Transfac:v7.0:M00395	ACCACct[c/g]c<br>19:47052568-47052576:+1	1.000/0.941	-		(N | N)
			Pax-2	Transfac:v7.0:M00098	t[c/g]ccCCAGCtaatgttttg<br>19:47052574-47052592:+1	0.992/0.930	0.992/0.929		(N | N)
			MyoD (myoblast determining factor)	Transfac:v7.0:M00184	acCAccT[C/G]cc<br>19:47052568-47052577:+1	3593.57/0.9	-/0.9	MotifScanner	(N | N)
			Ebox (E-box)	Transfac:v12.1:M01034	cCACCT[c/G]cCc<br>19:47052569-47052578:+1	0.998/0.996	-	MATCH (minFP)	(N | N)
**upstream**	**novel-4**<br>(+Pax-2, Msx-1)<br>(- 3 x Evi-1)	[G/T]	Pax-2	Transfac:v7.0:M00098	tatcCCACTgttt[g/t]taggt<br>19:47052881-47052899:+1	-	1.000/0.900	pMATCH (minSUM)	(N | N)
			Evi-1	Transfac:v7.0:M00081	tcccactgtTT[g/T]TAg<br>19:47052883-47052897:+1	0.898/0.838	-		(N | N)
			Msx-1	Transfac:v7.0:M00394	cacTGTTT[g/t]<br>19:47052886-47052894:+1	-	0.951/0.959		(N | N)
			Pax-2	Transfac:v7.0:M00098	cactGTTT[G/T]taggtactac<br>19:47052886-47052904:+1	0.971/0.951	0.974/0.952		(N | N)
			En-1	Transfac:v7.0:M00396	GTTT[g/T]ta<br>19:47052890-47052896:+1	1.000/0.990	-		(N | N)
			Evi-1	Transfac:v7.0:M00081	tt[g/t]taggtaCTACAg<br>19:47052892-47052906:+1	0.794/0.823	-		(N | N)
			Pax-2	Transfac:v7.0:M00098	[g/t]tagGTACTacagcctcaa<br>19:47052894-47052912:+1	0.903/0.899	0.903/0.898		(N | N)
**upstream**	**novel-5**<br>(+ Oct-1, HOXA3, CREB_Q2; Broad-complex_3, 671.634/0.9;)	[A/G]	Pax-2	Transfac:v7.0:M00098	gtatGATTCcaactat[a/g]tg<br>19:47053105-47053123:+1	0.992/0.935	0.992/0.923	pMATCH (minSUM)	(N | N)
			Evi-1	Transfac:v7.0:M00081	tatgattccAACTAt[a/g]<br>19:47053106-47053120:+1	0.782/0.842	0.782/0.842		(N | N)
			Evi-1	Transfac:v7.0:M00081	ttccaactaT[A/G]TGAc<br>19:47053111-47053125:+1	0.842/0.875	0.772/0.850		(N | N)
			v-Myb	Transfac:v7.0:M00003	tccAACTAt[a/g]<br>19:47053112-47053121:+1	1.000/0.982	1.000/0.968		(N | N)
			Msx-1	Transfac:v7.0:M00394	aacTAT[A/G]Tg<br>19:47053115-47053123:+1	0.909/0.931	0.917/0.936		(N | N)
			Pbx-1	Transfac:v7.0:M00096	actAT[A/G]TGa<br>19:47053116-47053124:+1	0.964/0.944	1.000/0.971		(N | N)
			Oct-1	Transfac:v7.0:M00162	ctat[a/g]tgaCATTCt<br>19:47053117-47053130:+1	-	0.964/0.971		(N | N)
			Pax-2	Transfac:v7.0:M00098	tat[a/g]TGACAttctggaaaa<br>19:47053118-47053136:+1	0.929/0.902	0.929/0.903		(N | N)
			Pax-2	Transfac:v7.0:M00098	at[a/g]tGACATtctggaaaaa<br>19:47053119-47053137:+1	0.992/0.899	0.992/0.912		(N | N)
			HOXA3	Transfac:v7.0:M00395	[A/g]TGACattc<br>19:47053121-47053129:+1	-	0.967/0.962		(N | N)
			Broad-complex_2	Jaspar:v3.0:MA0011	aaCTAt[a/g]t<br>19:47053115-47053122:+1	953.749/0.9	1187.41/0.9	MotifScanner	(N | N)
			Broad-complex_3	Jaspar:v3.0:MA0012	ccAACTAt[a/g]tg<br>19:47053113-47053123:+1	-/0.9	671.634/0.9<br>542.52/0.9		(N | N)
			CREB_Q2<br>(cyclic AMP response element-binding protein) (CCAAT-enhancer binding protein)	Transfac:v7.0:M00177	t[a/g]TGACaTtctg<br>19:47053120-47053131:+1	-/0.9	67.5249/0.9		(N | N)
**upstream**	**novel-6**<br>(+ Pax-2, YY!)<br>(- HOXA3)	[T/G]	p300	Transfac:v7.0:M00033	aactGTAGTacca[t/g]<br>19:47053609-47053622:+1	0.994/0.980	1.000/0.979	pMATCH (minSUM)	(N | N)
			YY1	Transfac:v7.0:M00059	tagtACCA[T/g]ctactcga<br>19:47053614-47053630:+1	-	0.998/0.990		(N | N)
			Pax-2	Transfac:v7.0:M00098	agtaCCA[T/G]Ctactcgacag<br>19:47053615-47053633:+1	0.992/0.909	0.974/0.899		(N | N)
			HOXA3	Transfac:v7.0:M00395	[t/G]CTACtcga<br>19:47053622-47053630:+1	0.987/0.964	-		(N | N)
			Pax-2	Transfac:v7.0:M00098	[t/g]actcGACAGgctgaggtag<br>19:47053623-47053641:+1	-	0.972/0.983	pMATCH (minFP)	(N | N)
**upstream**	**novel-7**<br>(+ HOXA3; Broad-complex_3, novel-5, 542.52/0.9;)<br>(- Evi-1, 2 x HOXA3, SRY)	[G/A]	Oct-1	Transfac:v7.0:M00137	cctGTAAAa[g/a]cta<br>19:47053892-47053904:+1	1.000/0.996	1.000/0.994	pMATCH (minSUM)	(N | N)
			Evi-1	Transfac:v7.0:M00081	tgtaaaa[g/a]cTAACAt<br>19:47053894-47053908:+1	0.851/0.821	0.851/0.850		(N | N)
			En-1	Transfac:v7.0:M00396	GTAAAa[g/a]<br>19:47053895-47053901:+1	0.996/0.990	-		(N | N)
			Evi-1	Transfac:v7.0:M00081	taaaa[g/a]ctaACATGt<br>19:47053896-47053910:+1	0.805/0.834	0.805/0.839		(N | N)
			HOXA3	Transfac:v7.0:M00395	AAA[g/A]Ctaac<br>19:47053898-47053906:+1	0.984/0.975	-		(N | N)
			SRY (sex-determining region Y)	Transfac:v7.0:M00148	AA[g/A]CTaa<br>19:47053899-47053905:+1	1.000/1.000	-		(N | N)
			Pax-2	Transfac:v7.0:M00098	a[g/a]ctAACATgtacaaaaat<br>19:47053900-47053918:+1	0.899/0.922	0.899/0.923		(N | N)
			Msx-1	Transfac:v7.0:M00394	a[g/a]cTAACAt<br>19:47053900-47053908:+1	0.949/0.940	0.949/0.940		(N | N)
			YY1	Transfac:v7.0:M00059	a[g/a]ctAACATgtacaaaa<br>19:47053900-47053916:+1	0.985/0.978	0.985/0.981		(N | N)
			HOXA3	Transfac:v7.0:M00395	[g/A]CTAAcatg<br>19:47053901-47053909:+1	0.951/0.944	-		(N | N)
			Msx-1	Transfac:v7.0:M00394	[g/a]ctAACATg<br>19:47053901-47053909:+1	0.962/0.967	0.962/0.957		(N | N)
			HOXA3	Transfac:v7.0:M00395	[G/a]CTAAcatg<br>19:47053901-47053909:+1	-	1.000/0.982		(N | N)
			Broad-complex_3	Jaspar:v3.0:MA0012	aAA[G/A]CTAacat<br>19:47053898-47053908:+1	953.749/0.9	-/0.9	MotifScanner	(N | N)
**upstream**	**novel-8**<br>(+ FOXJ2, Msx-1, Evi-1Pax-2, CdxA, YY1)<br>(- Pbx-1, 2 x Msx-1, v-Myb)	[G/A]	Pax-2	Transfac:v7.0:M00098	acatGTACAaaaatt[g/a]aca<br>19:47053905-47053923:+1	0.915/0.904	0.915/0.907	pMATCH (minSUM)	(N | N)
			FOXJ2 (fork head box J 2)	Transfac:v7.0:M00423	tgtaCAAAAatt[g/a]a<br>19:47053908-47053921:+1	-	0.968/0.891		(N | N)
			Evi-1	Transfac:v7.0:M00081	tacaaaaatT[G/A]ACAc<br>19:47053910-47053924:+1	0.851/0.867	0.921/0.892		(N | N)
			HOXA3	Transfac:v7.0:M00395	[G/A]ACAAAatt<br>19:47053911-47053919:+1	0.973/0.974	0.973/0.974		(N | N)
			Msx-1	Transfac:v7.0:M00394	caaAAATT[g/a]<br>19:47053912-47053920:+1	-	0.989/0.978		(N | N)
			Pbx-1a (homeo domain factor Pbx-1)	Transfac:v7.0:M00096	aaaAATT[g/A]a<br>19:47053913-47053921:+1	0.983/0.955	-		(N | N)
			HOXA3	Transfac:v7.0:M00395	AAAATt[g/a]ac<br>19:47053914-47053922:+1	0.970/0.964	0.970/0.964		(N | N)
			En-1	Transfac:v7.0:M00396	AAAATt[g/a]<br>19:47053914-47053920:+1	-	1.000/0.996		(N | N)
			Msx-1	Transfac:v7.0:M00394	aaaTT[g/A]ACa<br>19:47053915-47053923:+1	0.913/0.931	-		(N | N)
			Msx-1	Transfac:v7.0:M00394	aatT[g/A]ACAc<br>19:47053916-47053924:+1	0.949/0.948	-		(N | N)
			v-Myb	Transfac:v7.0:M00003	aatT[g/A]ACAcc<br>19:47053916-47053925:+1	1.000/0.977	-		(N | N)
			Pax-2	Transfac:v7.0:M00098	aatt[G/a]ACACcagggacaca<br>19:47053916-47053934:+1	-	1.000/0.920		(N | N)
			CdxA	Transfac:v7.0:M00100	ATT[G/a]Aca<br>19:47053917-47053923:+1	-	1.000/0.982		(N | N)
			YY1	Transfac:v7.0:M00059	t[g/a]acACCAGggacacag<br>19:47053919-47053935:+1	-	1.000/0.978		(N | N)
**upstream**	**novel-9**<br>(+ HOXA3, HLF, AP-2alpha, bZIP910, CREB, CREB1, CREBP1)<br>(- Msx-1, CdxA, POU3F2)	[C/T]	Msx-1	Transfac:v7.0:M00394	tgcCTTA[c/T]g<br>19:47054208-47054216:+1	0.917/0.943	-	pMATCH (minSUM)	(N | N)
			CdxA	Transfac:v7.0:M00100	CCTTA[c/t]g<br>19:47054210-47054216:+1	0.971/0.973	-		(N | N)
			HOXA3	Transfac:v7.0:M00395	CCTTA[c/t]gtc<br>19:47054210-47054218:+1	-	0.987/0.953		(N | N)
			Msx-1	Transfac:v7.0:M00394	cctTA[C/T]GTc<br>19:47054210-47054218:+1	0.955/0.933	0.992/0.958		(N | N)
			HOXA3	Transfac:v7.0:M00395	CTTA[C/T]gtcc<br>19:47054211-47054219:+1	0.970/0.959	0.957/0.948		(N | N)
			HLF	Transfac:v7.0:M00260	CTTA[C/t]gtcca<br>19:47054211-47054220:+1	-	0.939/0.953		(N | N)
			YY1	Transfac:v7.0:M00059	ta[c/t]gTCCATcaaaactc<br>19:47054213-47054229:+1	1.000/0.983	1.000/0.981		(N | N)
			Msx-1	Transfac:v7.0:M00394	a[c/t]gTCCATc<br>19:47054214-47054222:+1	0.902/0.933	0.902/0.933		(N | N)
			Pbx-1b (homeo domain factor Pbx-1)	Transfac:v7.0:M00124	ta[c/t]gTCcATCAAaac<br>19:47054213-47054227:+1	2253.81/0.9	10900.5/0.9	MotifScanner	(N | N)
			Oct-1 (octamer-binding factor 1, POU2F1)	Transfac:v7.0:M00161	ctgcTTtgCcTta[c/t]<br>19:47054202-47054215:+1	180.893/0.9	101.817/0.9		(N | N)
			CREB (deltaCREB)	Transfac:v7.0:M00113	tgccTtA[C/t]GTcc<br>19:47054208-47054219:+1	-/0.9	259.949/0.9		(N | N)
			AP-2alpha (Activator Protein-2)	Transfac:v7.0:M00469	GCCtta[c/t]Gt<br>19:47054209-47054217:+1	-/0.9	384.776/0.9		(N | N)
			CREB	Transfac:v7.0:M00039	TTA[C/T]GTCC<br>19:47054212-47054219:+1	-/0.9	149.021/0.9		(N | N)
			CREBP1	Transfac:v7.0:M00040	TTA[C/T]GTCC<br>19:47054212-47054219:+1	-/0.9	1153.47/0.9		(N | N)
			POU3F2 (N-Oct-5a, N-Oct-5b)	Transfac:v7.0:M00464	TTA[C/T]GtccAT<br>19:47054212-47054221:+1	137.922/0.9	-/0.9		(N | N)
			CREB1 (cyclic AMP response element-binding protein)	Jaspar:v3.0:MA0018	ttgccTtA[C/t]Gtc<br>19:47054207-47054218:+1	-/0.9	358.13/0.9		(N | N)
			AP-2alpha	Jaspar:v3.0:MA0003	GCCtta[c/t]Gt<br>19:47054209-47054217:+1	-/0.9	383.363/0.9		(N | N)
			bZIP910	Jaspar:v3.0:MA0096	cTtA[C/t]GT<br>19:47054211-47054217:+1	-/0.9	202.172/0.9		(N | N)
			Pbx1	Jaspar:v3.0:MA0070	a[c/t]gTCcATCAaa<br>19:47054214-47054225:+1	2378.14/0.9	8330.43/0.9		(N | N)
**upstream**	**novel-10**<br>(- C/EBPalpha)	[T/C]	Pax-6	Transfac:v7.0:M00097	ctctgCTTTAtataaatttta[t/c]<br>19:47054227-47054247:+1	0.948/0.968	0.948/0.968	pMATCH (minSUM)	(N | N)
			Oct-1	Transfac:v7.0:M00137	tatATAAAttta[t/c]<br>19:47054235-47054247:+1	1.000/0.995	1.000/0.995		(N | N)
			Evi-1	Transfac:v7.0:M00081	atataaattTTA[T/C]Ct<br>19:47054236-47054250:+1	0.819/0.832	0.898/0.861		(N | N)
			Pax-2	Transfac:v7.0:M00098	ataaATTTTa[t/c]ctaagctt<br>19:47054238-47054256:+1	0.982/0.932	0.982/0.941		(N | N)
			C/EBPalpha	Transfac:v7.0:M00116	atTTTA[T/c]ctaagct<br>19:47054242-47054255:+1	1.000/0.998	-		(N | N)
			CdxA	Transfac:v7.0:M00100	ATTTTa[t/c]<br>19:47054242-47054248:+1	0.979/0.981	0.979/0.972		(N | N)
			CdxA	Transfac:v7.0:M00100	TTTTA[t/c]c<br>19:47054243-47054249:+1	0.982/0.983	1.000/0.972		(N | N)
			Pax-2	Transfac:v7.0:M00098	ttta[T/C]CTAAgcttcactct<br>19:47054244-47054262:+1	0.924/0.907	0.949/0.922		(N | N)
			Msx-1	Transfac:v7.0:M00394	a[t/c]cTAAGCt<br>19:47054247-47054255:+1	0.989/0.968	0.989/0.967		(N | N)
			HOXA3	Transfac:v7.0:M00395	[T/C]CTAAgctt<br>19:47054248-47054256:+1	1.000/0.955	0.951/0.946		(N | N)
			box_2_2_TTACCTA	Model:EPD Vertebrate 3*	TTA[t/C]CTA<br>19:47054245-47054251:+1	17423.7	N.A.	MotifSampler	(N | N)
			box_2_2_mTGTTACCTA	Model:EPD Vertebrate 3*	ATTTTA[t/C]CTA<br>19:47054242-47054251:+1	61169.9	N.A.		(N | N)
**upstream**	**novel-11**<br>(+ Oct-1, Pax-2, SRY)<br>(- YY1)	[T/C]	Oct-1	Transfac:v7.0:M00137	cctGTAATc[t/c]cag<br>19:47054440-47054452:+1	-	1.000/0.996	pMATCH (minSUM)	(N | N)
			HOXA3	Transfac:v7.0:M00395	CCTGTaatc[t/c]<br>19:47054440-47054448:+1	0.970/0.958	0.970/0.958		(N | N)
			Pax-2	Transfac:v7.0:M00098	gtaaTC[T/c]CAgcattttggg<br>19:47054443-47054461:+1	-	0.989/0.931		(N | N)
			En-1	Transfac:v7.0:M00396	GTAATc[t/c]<br>19:47054443-47054449:+1	0.992/0.992	0.992/0.992		(N | N)
			YY1	Transfac:v7.0:M00059	taatC[t/C]CAGcattttgg<br>19:47054444-47054460:+1	0.996/0.973	-		(N | N)
			SRY	Transfac:v7.0:M00148	AATC[T/c]ca<br>19:47054445-47054451:+1	-	1.000/0.976		(N | N)
			Pax-2	Transfac:v7.0:M00098	aatc[T/C]CAGCattttgggag<br>19:47054445-47054463:+1	0.992/0.952	0.966/0.937		(N | N)
			Pax-2	Transfac:v7.0:M00098	atc[t/c]CAGCAttttgggagg<br>19:47054446-47054464:+1	0.918/0.927	0.918/0.927		(N | N)
			YY1	Transfac:v7.0:M00059	tc[t/c]cAGCATtttgggag<br>19:47054447-47054463:+1	0.998/0.990	0.998/0.988		(N | N)
**upstream**	**rs930102**<br>(- Pax-2, HOXA2, Evi-1)	[C/T]	Pax-2	Transfac:v7.0:M00098	cgagG[c/T]GGCagggccggac<br>19:47056230-47056248:+1	0.914/0.926	-	pMATCH (minSUM)	(Y | N)
			HOXA3	Transfac:v7.0:M00395	CGAGG[c/t]ggc<br>19:47056230-47056238:+1	0.973/0.979	-		(Y | N)
			COMP1	Transfac:v7.0:M00057	cgagg[c/t]GGCAGggccggacgccga<br>19:47056230-47056253:+1	0.929/0.834	0.929/0.834		(Y | N)
			En-1	Transfac:v7.0:M00396	GAGG[c/T]gg<br>19:47056231-47056237:+1	0.992/0.988	-		(Y | N)
			Pax-2	Transfac:v7.0:M00098	agg[c/t]GGCAGggccggacgc<br>19:47056232-47056250:+1	0.887/0.901	0.887/0.902		(Y | N)
			Sp1 (stimulating protein 1)	Transfac:v7.0:M00008	g[c/t]GGCAGggc<br>19:47056234-47056243:+1	1.000/0.976	1.000/0.976		(Y | N)
			Pax-5 (B-cell-specific activating protein)	Transfac:v7.0:M00143	accgggcgCgagG[c/t]<br>gGcagggccggacg<br>19:47056222-47056249:+1	2609.34/0.9	1057.56/0.9	MotifScanner	(y+ | N)
**upstream**	**novel-12**<br>(+ 2 x Sp1, Pax-5, AP-2alpha)<br>(- Sp1, Pax-2)	[G/C]	Sp1	Transfac:v7.0:M00008	ccGG[g/C]GCggg<br>19:47056289-47056298:+1	1.000/0.949	-	pMATCH (minSUM)	(y | N)
			Sp1	Transfac:v7.0:M00008	cgG[G/c]GCGggc<br>19:47056290-47056299:+1	-	0.957/0.956		(y | N)
			Sp1	Transfac:v7.0:M00008	gg[G/c]GCGGgcc<br>19:47056291-47056300:+1	-	1.000/0.985		(y+ | N)
			Pax-2	Transfac:v7.0:M00098	gg[g/c]gCGGGCcgctccgcc<br>19:47056291-47056309:+1	0.938/0.891	-		(y+ | N)
			AP-2alpha	Jaspar:v3.0:MA0003	GCCcgg[g/c]gc<br>19:47056287-47056295:+1	53.929/0.9	170.435/0.9	MotifLocator	(y- | N)
			Pax-5	Transfac:v7.0:M00144	cccgaGgcgcccgg[g/c]<br>GCGggcCcgtccc<br>19:47056279-47056306:+1	84.2745/0.9	379.43/0.9	MotifScanner	(y- | N)
			Pax-5	Transfac:v12.1:M00144	cccgaggcgcccgG[G/C]<br>GCGggcccgtccc<br>19:47056279-47056306:+1	0.839/0.753	0.873/0.767	MATCH (minFP)	(y| N)
			AP-2alpha	Transfac:v11.2:M00469	GCCCGg[g/c]gc<br>19:47056287-47056295:+1	-	1.000/1.000		(y- | N)
**upstream**	**novel-13**<br>(+AP-2alpha; Eip74EF; PU.1/Spi-1; Pax-2; c-Ets-1/p54; v-Myb)<br>(- AP-2alpha)	[A/G]	AP-2alpha	Jaspar:v7.0:MA0003	GCCgga[a/g]cc<br>19:47056536-47056544:+1	-/0.9	31.5705/0.9	MotifLocator	(Y | N)
			Eip74EF	Jaspar:v7.0:MA0026	ccGGA[A/g]c<br>19:47056537-47056543:+1	-/0.9	394.501/0.9		(Y | N)
			PU.1 (Spi-1)	Jaspar:v7.0:MA0080	cGGA[A/g]c<br>19:47056538-47056543:+1	-/0.9	311.828/0.9		(Y | N)
			AP-2alpha	Jaspar:v3.0:MA0003	[A/G]CCcggcgt<br>19:47056542-47056550:+1	41.181/0.9	-/0.9		(y- | N)
			Pax-2	Transfac:v7.0:M00098	ggagCCGGA[a/g]cccggcgtt<br>19:47056533-47056551:+1	-	0.969/0.893	pMATCH (minSUM)	(y | N)
			c-Ets-1 (p54)	Transfac:v7.0:M00032	gCCGGA[a/g]ccc<br>19:47056536-47056545:+1	-	1.000/0.982		(Y | N)
			COMP1	Transfac:v7.0:M00057	ccgga[a/g]CCCGGcgttgaaggggcc<br>19:47056537-47056560:+1	0.807/0.852	0.807/0.844		(y- | N)
			v-Myb	Transfac:v7.0:M00003	cggA[A/g]CCCgg<br>19:47056538-47056547:+1	-	0.972/0.971		(y | N)
			p300	Transfac:v7.0:M00033	[a/g]cccGGCGTtgaag<br>19:47056542-47056555:+1	1.000/0.979	1.000/0.979		(y- | N)
**upstream**	**novel-14**<br>(+ HOXA3, SRY)<br>(- Sp1)	[A/G]	COMP1	Transfac:v7.0:M00057	ccggagCCCGGcgttgaagg[a/g]gcc<br>19:47056537-47056560:+1	0.807/0.852	0.807/0.844	pMATCH (minSUM)	(y | N)
			Pax-2	Transfac:v7.0:M00098	cggcGTTGAagg[a/g]gccgtg<br>19:47056545-47056563:+1	0.982/0.935	0.982/0.939		(y | N)
			Pax-2	Transfac:v7.0:M00098	ggcgTTGAAgg[a/g]gccgtgg<br>19:47056546-47056564:+1	0.955/0.930	0.955/0.941		(y- | N)
			Msx-1	Transfac:v7.0:M00394	cgtTGAAGg[a/g]<br>19:47056548-47056556:+1	0.963/0.938	0.963/0.938		(y- | N)
			HOXA3	Transfac:v7.0:M00395	CGTTGaagg[a/g]<br>19:47056548-47056556:+1	1.000/0.976	1.000/0.976		(y- | N)
			HOXA3	Transfac:v7.0:M00395	TTGAAgg[a/g]g<br>19:47056550-47056558:+1	-	0.973/0.943		(y- | N)
			HOXA3	Transfac:v7.0:M00395	TGAAGg[a/g]gc<br>19:47056551-47056559:+1	1.000/0.954	1.000/0.954		(y- | N)
			SRY	Transfac:v7.0:M00148	AAGG[A/g]gc<br>19:47056553-47056559:+1	-	1.000/0.975		(y- | N)
			Sp1	Transfac:v7.0:M00008	gg[a/G]GCCGtgg<br>19:47056555-47056564:+1	0.981/0.942	-		(y | N)
			AP-2alpha	Jaspar:v3.0:MA0003	[a/g]GCCgtggga<br>19:47056558-47056566:+1	51.4922/0.9	40.4248/0.9	MotifLocator	(y | N)
**upstream**	**novel-15**<br>(+ YY1, Evi-1, Pbx-1)<br>(- 2 x Pax-2, Kid3, v-Myb, Tst-1, GR-a)	[A/G]	COMP1	Transfac:v7.0:M00057	aagtaaCGGGGggtaccac[a/g]gttt<br>19:47056566-47056589:+1	0.807/0.842	0.807/0.842	pMATCH (minSUM)	(y- | N)
			COMP1	Transfac:v7.0:M00057	agtaacGGGGGgtaccac[a/g]gttta<br>19:47056567-47056590:+1	0.893/0.832	0.893/0.832		(y- | N)
			p300	Transfac:v7.0:M00033	acggGGGGTaccac[a/g]<br>19:47056571-47056584:+1	1.000/0.997	1.000/0.997		(y- | N)
			Pax-2	Transfac:v7.0:M00098	ggggGTACCac[a/g]gtttagg<br>19:47056574-47056592:+1	0.895/0.891	-		(y- | N)
			YY1	Transfac:v7.0:M00059	gtacCAC[A/g]Gtttaggat<br>19:47056578-47056594:+1	-	0.996/0.980		(y- | N)
			Evi-1	Transfac:v7.0:M00081	taccac[a/g]gtTTAGGa<br>19:47056579-47056593:+1	-	0.819/0.822		(y- | N)
			Pbx-1	Transfac:v7.0:M00096	accAC[A/g]GTt<br>19:47056580-47056588:+1	-	0.930/0.937		(y- | N)
			HOXA3	Transfac:v7.0:M00395	C[A/G]GTTtagg<br>19:47056584-47056592:+1	0.956/0.953	0.940/0.941		(y- | N)
			Msx-1	Transfac:v7.0:M00394	c[a/g]gTTTAGg<br>19:47056584-47056592:+1	0.940/0.959	0.940/0.959		(y- | N)
			HOXA3	Transfac:v7.0:M00395	ACCAC[a/g]gtt<br>19:47056580-47056588:+1	1.000/0.995	1.000/0.995	pMATCH (minSUM, minFP)	(y+ | N)
			Pax-2	Jaspar:v3.0:MA0067	tacCAc[a/g]g<br>19:47056579-47056586:+1	112.092/0.9	-/0.9	MotifLocator	(Y | N)
			v-Myb	Transfac:v7.0:M00227	taccAC[A/G]Gt<br>19:47056579-47056587:+1	96.1399/0.9	-/0.9	MotifScanner	(y+ | N)
			Tst-1 (POU-factor Tst-1/Oct-6)	Transfac:v7.0:M00133	cac[a/g]gtTtaggatgc<br>19:47056582-47056596:+1	181.713/0.9	-/0.9		(y- | N)
			GR-a (Glucocorticoid response el.)	Transfac:v7.0:M00205	[a/g]gtttaggatGcgCTg<br>19:47056585-47056600:+1	1248.57/0.9	-/0.9		(y- | N)
			Spz1 (spermatogenic Zip)	Transfac:v7.0:M00446	aacGGgGGgtaccac[A/G]<br>19:47056570-47056584:+1	705.638/0.9	705.638/0.9		(y | N)
			Ik-2 (Ikaros 2)	Transfac:v7.0:M00087	[A/G]gttTaGGAtgcgctg<br>19:47056586-47056597:+1	591.664/0.9	359.408/0.9		(y- | N)
			Spz1	Transfac:v7.0:M00446	aacGGGGGgtaccac[a/g]<br>19:47056570-47056584:+1	705.638/0.9	705.638/0.9		(y- | N)
			GR-alpha<br>(Glucocorticoid response element)	Transfac:v7.0:M00205	[a/g]gtttAggaTGcgCTg<br>19:47056585-47056600:+1	170.47/0.9	-/0.9		(y- | N)
			Spz1	Transfac:v12.1:M00446	aacGGGGGgtaccac[a/g]<br>19:47056570-47056584:+1	0.998/0.962	0.998/0.962	MATCH (minFP)	(y- | N)
			Kid3 (ZNF354C)	Transfac:v12.1:M01160	CCAC[a/g]<br>19:47056581-47056585:+1	1.000/1.000	-		(Y | N)
**2nd intron**	**novel-16**	[CC/C]	box_2_1_GGTyCTAG	Model:EPD Human 3*	GGTC[CC/C]TGG<br>19:47056832-47056839:+1	4788.67	N.A.	MotifSampler	(N | N)
			box_2_1_GGGCCTnCs	Model:EPD Vertebrate 3*	GGTC[CC/C]TGGC<br>19:47056832-47056840:+1	2321.43	N.A.		(N | N)
	**rs2075749**	[C/G]	Sp1	Transfac:v7.0:M00008	[c/g]GAGGCTTGTT<br>19:47056845-47056854:+1	111.864/0.3	188.282/0.3	MotifScanner	(Y | N)
	**novel-17**	[-/T]	box_1_2_CTGnmTTG	Model:EPD Vertebrate 3*	CTGTTT[-/T]G<br>19:47057006-47057013:+1	1441.2	N.A.	MotifSampler	(y- | N)
			box_1_1_TGTnTTsnGG	Model:EPD Human 3*	TGTTT[-/T]GGTC<br>19:47057007-47057016:+1	1385.25	N.A.		(y | N)
			box_1_1_TTGGnCnTA	Model:EPD Vertebrate 3*	T[-/T]GGTCTTA<br>19:47057011-47057019:+1	37103.9	N.A.		(Y | N)
			box_1_2_GGnCTkAG	Model:EPD Human 3*	[-/t]GGTCTTAG<br>19:47057013-47057020:+1	10389.1	N.A.		(Y | N)
			box_2_1_GGTyCTAG	Model:EPD Human 3*	[-/t]GGTCTTAG<br>19:47057013-47057020:+1	12925.1	N.A.		(Y | N)
**4th intron**	**rs1366610**<br>(- Sp1, MZF1_5-13, Mzf1;)<br>(+ Spz1; Zic1; Zic2; Olf-1, novel-42, 2012.2/0.9; E47, 5494.35/0.9; HSF1, novel-41, 613.405/0.9; HSF2, novel-42, 630.919/0.9;)	[A/G]	Szf1-1	Transfac:v11.2:M01109	gCAGGGtag[a/g]ggggg<br>19:47065129-47065143:+1	1.000/0.815	1.000/0.818	MATCH (minFP)	(y | N)
			Spz1 (spermatogenic Zip)	Transfac:v7.0:M00446	taaGcaGGgtag[a/g]gg<br>19:47065126-47065140:+1	-/0.5	201.793/0.5	MotifScanners	(y- | N)
			Mzf1	Transfac:v7.0:M00084	agggtAG[a/G]GGggg<br>19:47065131-47065143:+1	201.027/0.5	-/0.5		(y | N)
			MZF1_5-13	Jaspar:v3.0:MA0057	gtAg[a/G]GGggg<br>19:47065134-47065143:+1	1253.7/0.5	-/0.5		(y- | N)
			Sp1	Jaspar:v3.0:MA0079	ag[a/G]Gggggct<br>19:47065136-47065145:+1	104.692/0.5	-/0.5		(y- | N)
			Zic3 (zinc finger protein of the cerebellum 3)	Transfac:v7.0:M00450	aG[a/G]GgGGgc<br>19:47065136-47065144:+1	-/0.4	48.1223/0.4		(y- | N)
	**novel-41**<br>(- MZF1_5-13, Mzf1, Sp1;)<br>(+ Zic1; Zic2; Olf-1, novel-42, 2012.2/0.9; Spz1. rs1366610, 526.149/0.9; E47, novel-42, 5494.35/0.9; HSF1, novel-42, 613.405/0.9; HSF2, novel-42, 630.919/0.9;)	[C/G]	Szf1-1	Transfac:v11.2:M01109	gCAGGGtaggggg[c/g]g<br>19:47065129-47065143:+1	1.000/0.815	1.000/0.823	MATCH (minFP)	(y | N)
			Mzf1	Transfac:v7.0:M00084	agggtAGGGGg[c/g]g<br>19:47065131-47065143:+1	201.027/0.5	-/0.5	MotifScanner	(y | N)
			MZF1_5-13	Jaspar:v3.0:MA0057	gtAgGGGg[c/g]g<br>19:47065134,47065143:+1	1253.7/0.5	-/0.5		(y- | N)
			Sp1	Jaspar:v3.0:MA0079	agGGgg[c/g]gct<br>19:47065136-47065145:+1	104.692/0.5	-/0.5		(y- | N)
			Zic3	Transfac:v7.0:M00450	aGGGgG[c/G]gC<br>19:47065136-47065144:+1	-/0.4	48.1223/0.4		(y- | N)
	**novel-42**<br>(+ HOXA3; E47, rs2075750, 5494.35/0.1; Spz1, rs1366610,; Zic3, novel-41)<br>(-Olf-1, HSF1, HSF2)	[T/G]	Olf-1 (olfactory neuron-specific factor)	Transfac:v7.0:M00261	atCaAtTCCCcAac[t/G]Aatggtc<br>[illegible]	7791.08/0.1	-/0.1	MotifScanner	(y | N)
			HOXA3	Transfac:v7.0: M00395	ac[T/g]Aatggt<br>[illegible]	-/0.4	121.962/0.4		(y- | N)
			HSF1 (heat shock factor 1)	Transfac:v7.0:M00146	c[t/G]AAtggTCc<br>19:47065189-47065198:+1	644.977/0.4	-/0.4		(y- | N)
			HSF2 (heat shock factor 2)	Transfac:v7.0:M00147	c[t/G]AAtggTCc<br>19:47065189-47065198:+1	654.993/0.4	-/0.4		(y- | N)
			Zic1 (zinc finger protein of the cerebellum 1)	Transfac:v7.0:M00448	c[t/G]aaTGGTC<br>19:47065189-47065197:+1	-/0.5	121.655/0.5		(y- | N)
			Zic2 (zinc finger protein of the cerebellum 2)	Transfac:v7.0:M00449	c[t/G]aaTGGTC<br>19:47065189-47065197:+1	-/0.5	49.014/0.5		(y- | N)
			box_2_2_yCCCAAsG	Model:EPD Vertebrate 3*	CCCCAAC[T/G]<br>19:47065183-47065190:+1	18227.9	N.A.	MotifSampler	(Y | N)
			box_1_1_CnAACGA	Model:EPD Human 3*	CCAAC[T/G]A<br>19:47065185-47065191:+1	2477.93	N.A.		(Y | N)
			box_1_2_AACGA	Model:EPD Vertebrate 3*	AAC[T/G]A<br>19:47065187-47065191:+1	2035.61	N.A.		(Y | N)
			box_2_2_CCAAGGGTm	Model:EPD Vertebrate 3*	C[T/G]AATGGTC<br>19:47065189-47065197:+1	31751.6	N.A.		(y- | N)
	**rs2075750**<br>(-Olf-1, novel-42)	[C/T]	E47	Transfac:v7.0:M00002	gcgGCAGGTGgctt[c/t]<br>19:47065505-47065519:+1	4874.7/0.1	5494.35/0.1<br>7751.3/0.1	MotifScanner	(Y | N)
			NF-1 (myogenin / NF-1 myogenin / nuclear factor 1 or related factors)	Transfac:v11.2:M00056	tt[c/t]<br>ttgagaagcCTGGCtcacagccagga<br>19:47065517,47065545:+1	-	0.900/0.713	MATCH (minFP)	(Y | N)
