# Supplementary material for: Targeted Resequencing and Analysis of the Diamond-Blackfan Anemia Disease Locus RPS19
Source: PLoS One. 2009 Jul 9;4(7):e6172. doi: 10.1371/journal.pone.0006172 (PMC2703794; doi:10.1371/journal.pone.0006172)
Supplement: Table S2 — Primer sequences for analysis of 5′upstream region. (0.16 MB PDF) [file pone.0006172.s004.pdf]

Table S2: Primer sequences for analysis of 5'upstream region

Name	Sequence[a]	Binding to sequence[b] start	stop
OE56 F	GCCACTGAGATTTGGAGAGTG	47048065	47048085
OE56 R	GTGAAGCCGACTTTGGTCTC	47049580	47049599
(b) F	ACCCTTAGAAGGGGCTCAAG	47049362	47049381
(b) R	GGCAGCATTGGAGTGTGTTA	47051000	47051019
UCR F	CCATCTATGTCTCCCCCTCA	47050860	47050879
UCR R	TCCCAAAGTTCCGAGATCAG	47051435	47051454
OE12 F	AAGCGATTCTCATGCCTCAT	47052314	47053333
OE12 R	TCTGCAAGTTTTGGCAAATG	47053276	47053295
NOVEL1 F	ACTGCACTCCAGAGCAGGAC	47053995	47054014
NOVEL1 R	ACCCATTTATCCCTCCATCC	47054195	47054214
NOVEL12 F	GCAGCCTTCCTCAAAAAGTG	47056740	47056759
NOVEL12R	CCCTCCCCTCAACACAACTA	47056936	47056955

[a]5' to 3' direction

[b]coordinates on chromosome 19 (hg18)

Barrio_table S2
