# Supplementary material for: Targeted Resequencing and Analysis of the Diamond-Blackfan Anemia Disease Locus RPS19
Source: PLoS One. 2009 Jul 9;4(7):e6172. doi: 10.1371/journal.pone.0006172 (PMC2703794; doi:10.1371/journal.pone.0006172)
Supplement: Figure S1 — Detailed view of the analyzed region. Every *.gff file available has been imported into the UCSC Genome Browser (Kuhn et al, 2009) for visualisation (see supplementary text S2). (1.71 MB PDF) [file pone.0006172.s001.pdf]

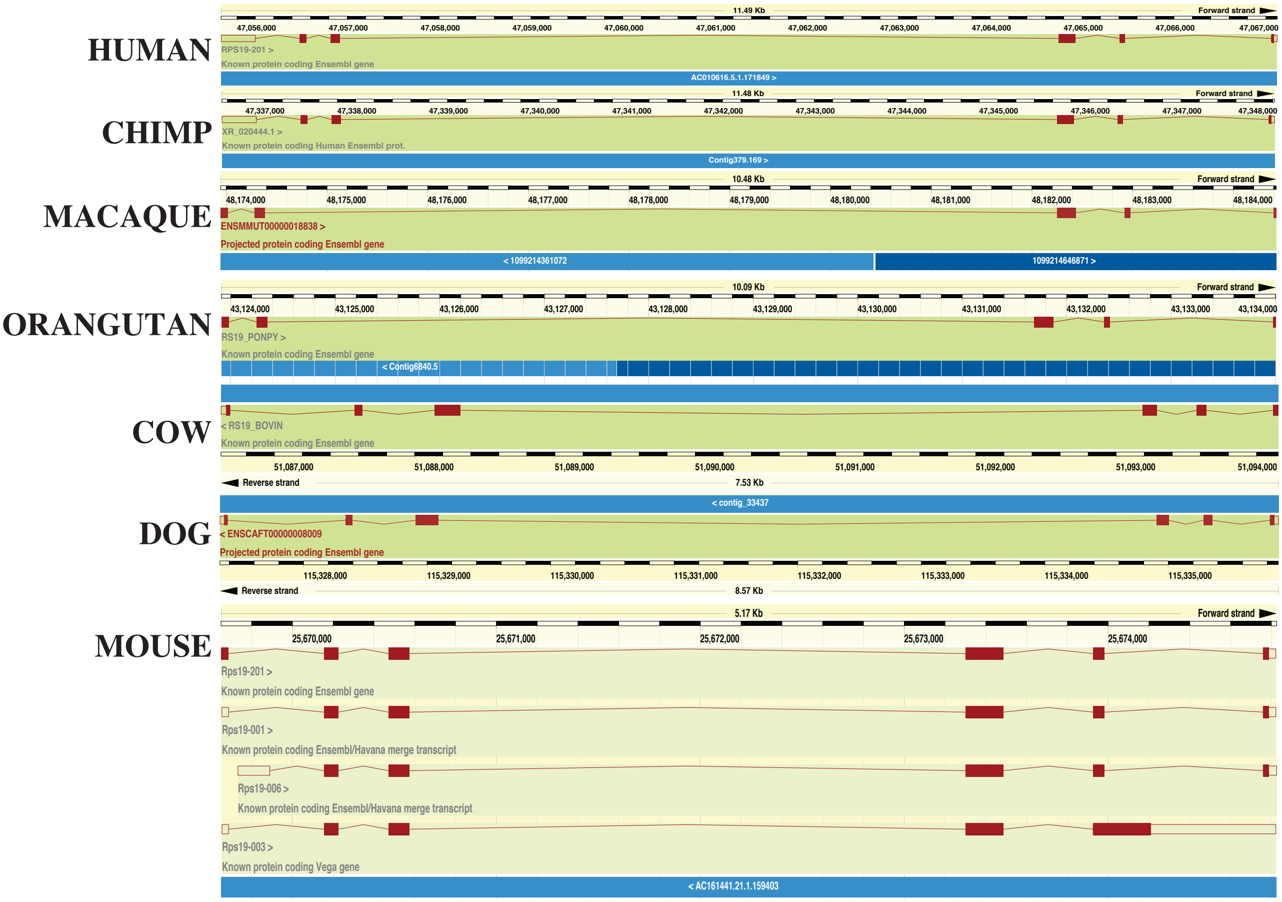

Barrio_Supplementary Figure S1
